# Iron deficiency is related to lower muscle mass in community‐dwelling individuals and impairs myoblast proliferation

**DOI:** 10.1002/jcsm.13277

**Published:** 2023-06-30

**Authors:** Joanna Sophia J. Vinke, Alan R. Gorter, Michele F. Eisenga, Wendy A. Dam, Peter van der Meer, Jacob van den Born, Stephan J.L. Bakker, Martijn F. Hoes, Martin H. de Borst

**Affiliations:** ^1^ Departments of Nephrology University Medical Center Groningen, University of Groningen Groningen The Netherlands; ^2^ Department of Cardiology University Medical Center Groningen, University of Groningen Groningen The Netherlands; ^3^ Department of Clinical Genetics Maastricht University Medical Center+ Maastricht The Netherlands; ^4^ CARIM School for Cardiovascular Diseases Maastricht The Netherlands

**Keywords:** Skeletal muscle, Iron

## Abstract

**Background:**

Loss of muscle mass is linked with impaired quality of life and an increased risk of morbidity and premature mortality. Iron is essential for cellular processes such as energy metabolism, nucleotide synthesis and numerous enzymatic reactions. As the effects of iron deficiency (ID) on muscle mass and function are largely unknown, we aimed to assess the relation between ID and muscle mass in a large population‐based cohort, and subsequently studied effects of ID on cultured skeletal myoblasts and differentiated myocytes.

**Methods:**

In a population‐based cohort of 8592 adults, iron status was assessed by plasma ferritin and transferrin saturation, and muscle mass was estimated using 24‐h urinary creatinine excretion rate (CER). The relationships of ferritin and transferrin saturation with CER were assessed by multivariable logistic regression. Furthermore, mouse C2C12 skeletal myoblasts and differentiated myocytes were subjected to deferoxamine with or without ferric citrate. Myoblast proliferation was measured with a colorimetric 5‐bromo‐2′‐deoxy‐uridine ELISA assay. Myocyte differentiation was assessed using Myh7‐stainings. Myocyte energy metabolism, oxygen consumption rate and extracellular acidification rate were assessed using Seahorse mitochondrial flux analysis, and apoptosis rate with fluorescence‐activated cell sorting. RNA sequencing (RNAseq) was used to identify ID‐related gene and pathway enrichment in myoblasts and myocytes.

**Results:**

Participants in the lowest age‐ and sex‐specific quintile of plasma ferritin (OR vs middle quintile 1.62, 95% CI 1.25–2.10, *P* < 0.001) or transferrin saturation (OR 1.34, 95% CI 1.03–1.75, *P* = 0.03) had a significantly higher risk of being in the lowest age‐ and sex‐specific quintile of CER, independent of body mass index, estimated GFR, haemoglobin, hs‐CRP, urinary urea excretion, alcohol consumption and smoking status. In C2C12 myoblasts, deferoxamine‐induced ID reduced myoblast proliferation rate (*P*‐trend <0.001) but did not affect differentiation. In myocytes, deferoxamine reduced myoglobin protein expression (−52%, *P* < 0.001) and tended to reduce mitochondrial oxygen consumption capacity (−28%, *P* = 0.10). Deferoxamine induced gene expression of cellular atrophy markers *Trim63* (+20%, *P* = 0.002) and *Fbxo32* (+27%, *P* = 0.048), which was reversed by ferric citrate (−31%, *P* = 0.04 and −26%, *P* = 0.004, respectively). RNAseq indicated that both in myoblasts and myocytes, ID predominantly affected genes involved in glycolytic energy metabolism, cell cycle regulation and apoptosis; co‐treatment with ferric citrate reversed these effects.

**Conclusions:**

In population‐dwelling individuals, ID is related to lower muscle mass, independent of haemoglobin levels and potential confounders. ID impaired myoblast proliferation and aerobic glycolytic capacity, and induced markers of myocyte atrophy and apoptosis. These findings suggest that ID contributes to loss of muscle mass.

## Introduction

Skeletal muscle mass is a key determinant of physical condition and mobility.^1^ Reduced muscle mass, or sarcopenia, is strongly linked with impaired quality of life and premature mortality, fuelling the need for modifiable risk factors.^1^, ^2^ Several factors may promote sarcopenia, including physical inactivity, reduced mobility and poor physical endurance.^1^, ^2^ Iron deficiency (ID) may adversely influence muscle strength, exercise capacity and endurance in chronically ill individuals and the elderly, but so far, it is unknown whether there is a relationship between ID and muscle mass in the general population.

ID is among the most common mineral deficiencies. The largest amount of iron in the body is used for haemoglobin production in the bone marrow and ID is a common cause of anaemia. In recent years, there has been increasing interest in implications of ID beyond erythropoiesis. Iron plays a role in deoxyribonucleic acid (DNA) synthesis and is essential to the function of numerous enzymes. Moreover, iron is a key component of myoglobin, responsible for oxygen storage in muscle cells. Finally, iron is crucial for mitochondrial function and aerobic metabolism, being involved in the citric acid cycle as well as the electron transport chain.^3^, ^4^

The relationship between ID and muscle cell function has been studied in cardiomyocytes and skeletal muscle cells. In cardiomyocytes, ID impairs oxidative phosphorylation and contractile function.^5^, ^6^ ID reduced left ventricular function in rodents.^7^, ^8^ In heart failure patients, ID has been associated with worse skeletal muscle strength and increased lactate production after exercise, while iron supplementation improves aerobic glycolysis.^9^, ^10^, ^11^ Also, in skeletal muscle cells, ID may influence energy metabolism.^12^, ^13^, ^14^ In humans, anaemia has been linked with reduced muscle mass,^15^ but data about the association between ID and muscle mass are scarce. Moreover, little is known about potential mechanisms by which ID might be related to reduced muscle mass.

Therefore, in the current study, we addressed whether ID is related to reduced muscle mass in the general population. Moreover, we investigated whether ID influences proliferation and differentiation rate, aerobic metabolism and cell viability in cultured mouse C2C12 myoblasts and differentiated myocytes.

## Methods

### Iron deficiency and muscle mass in community‐dwelling individuals

We used cross‐sectional data from the Prevention of Renal and Vascular End‐stage Disease (PREVEND) study, a prospective, population‐based cohort of Dutch community‐dwelling individuals aged 25 to 75 years. The study was approved by the local ethics committee and complied with the principles of the Declaration of Helsinki. A full description of the methods, including extensive description of the statistical methods, is provided in the supplemental material. Written informed consent was obtained from all participants before enrolment. In this study, 8592 participants were enrolled at baseline. For the current analysis, we excluded participants with missing data on urinary creatinine excretion rate (CER) or ferritin (Figure S1). All participants were asked to collect two consecutive 24‐h urine samples before their visit to the clinic, of which the average was calculated. To limit potential 24‐h urine collection errors, we excluded participants at the lowest or highest 2.5% of difference between expected and measured 24‐h urine volume,^16^ leaving 5571 participants available for analysis. Fasting blood samples and 24‐h urine samples were collected at the same study visit. Transferrin saturation (TSAT) was calculated as 100 × plasma iron (μmol/L)/(plasma transferrin (g/L) × 25). The average CER of the two 24‐h urine samples was used as a parameter for muscle mass. CER was divided into quintiles stratified for age and sex, two major factors influencing muscle mass. The odds ratio of being in the lowest quintile of age‐ and sex‐stratified CER was compared between participants across quintiles of age‐ and sex‐stratified ferritin and TSAT levels, using logistic regression (model 1). Upon multivariable analysis (model 2), we adjusted for estimated glomerular filtration rate (eGFR), body mass index (BMI), high‐sensitive C‐reactive protein (hs‐CRP), 24‐h urinary urea excretion (reflecting protein intake), alcohol consumption and smoking status. In model 3, we additionally adjusted for plasma haemoglobin (Hb). In sensitivity analyses, we substituted CER for length‐indexed CER^17^ and length^2‐indexed CER,^18^ given that muscle mass depends on body size. Furthermore, we repeated the analyses after exclusion of the 5% most extreme ferritin or TSAT values by calculating absolute differences from the median.

### Cell culture

Mouse C2C12 skeletal myoblasts (ATCC, Cat. no. CRL‐1772) were cultured in Dulbecco's Modified Eagle Medium (DMEM, Gibco Cat. No. 41966‐029) supplemented with 10% fetal bovine serum (FBS, Sigma‐Aldrich Cat. No. F7524) and 1% penicillin–streptomycin (ps; 5000 U/mL penicillin, 5000 μg/mL streptomycin, Gibco) using T25 flasks (Thermo Scientific Cat. No. 156367). Cells were incubated at 37 °C, 5% CO_2_ (Thermo Scientific Forma Series II Water Jacket CO2 incubator) and 100% humidity. Sub‐culturing and refreshing medium was performed three times per week. For sub‐culturing, cells were washed with phosphate buffered saline (PBS; Biowhittaker Cat. No. 15‐512Q) before trypsinization with 0.05% Trypsin–EDTA (Thermo Scientific, Cat. No. 15400‐054). Collected cells were then diluted for sub‐culturing or counted for seeding on plate. Counting of living cells was done using a trypan blue staining and a Bürker cell counting chamber (Marienfeld‐superior Cat. No. 0640211). For the differentiation from myoblasts to myocytes, cell culture was grown to a confluence of >70% in cell culture plates before switching from culture medium to differentiation medium (DMEM, 2.5%; horse serum (HS, Sigma‐Aldrich; Merck KgaA Cat. No. H1138), 1% ps). After seven days, this resulted in the gene expression of differentiation markers (Myh7, Myod, Myog, Figure S2) and formation of skeletal myotubes across the plate and experimental intervention was initiated for up to three days.

### Induction of iron deficiency and iron repletion

To induce ID during cell culture, deferoxamine (DFO; Sigma‐Aldrich Cat. No. D9533) was added to the medium. Unless stated otherwise, a DFO concentration of 7.5 μM was used. Repletion of iron to the cells was achieved by supplementing medium containing DFO with ferric citrate (FC; Sigma‐Aldrich Cat. No. F3388). Unless stated otherwise, a FC concentration of 10 μM was used. The cells were typically incubated with or without DFO and FC for three days, except for production of quantitative polymerase chain reaction (qPCR) samples for which they were incubated one day.

### Assays

Experimental methods are described in brief below. A more extensive description is provided in the Supplemental material.

Proliferation rate of C2C12 myoblasts was determined using a colorimetric 5‐bromo‐2′‐deoxy‐uridine (BrdU) cell proliferation ELISA kit (Abcam, Cat. No. ab26556).

Differentiation rate of C2C12 myoblasts was determined using staining techniques. The fusion index, or the percentage of nuclei being in a differentiated cell, was counted.

Oxygen consumption rate (OCR) and extracellular acidification rate (ECAR) of skeletal myocytes were measured using a Seahorse Mito Stress test. During the assay, three baseline measurements were taken, after which ATP synthase inhibitor Oligomycin A was injected into the wells to inhibit complex I of the oxidative phosphorylation process. Then, carbonyl cyanide 4‐(trifluoromethoxy)‐phenylhydrazone (FCCP) was injected to uncouple the oxidative phosphorylation and induce rapid oxidation of energy substrates. All data were acquired using a Seahorse XF24 or XF96 analyser and normalized for total protein in the well. To assess ATP‐linked respiration, the decrease in oxygen consumption rate after the injection of oligomycin A was calculated (OCR_basal_ − OCR_oligomycin_). To assess respiratory reserve of the cells, the increase in oxygen consumption after injection of FCCP compared with baseline was calculated (OCR_FCCP_ − OCR_basal_).

For western blotting, C2C12 cell lysate was collected and total protein concentration measured using the Bicinchoninic acid (BCA) protein assay (ThermoScientific, Cat. No. 23227). Using normalized protein amounts, ferritin heavy chain I (Fth) and myoglobin bands were visualized and analysed after normalization for β‐actin.

For quantitative real‐time polymerase chain reaction (PCR) and for ribonucleic acid (RNA) sequencing, total RNA was isolated from C2C12 myoblasts and myocytes. Quantitative PCR (qPCR) technology was then performed by mixing collected cDNA with primers for target genes *Fbxo32*, *Trim63*, *Becn1*, *TfR*, *Slc39a14*, *Slc40a1*, *Myh7*, *Myod*, *Myog*, *Bax*, *Bcl2*, *Casp9* and *Rplp0* (Table S1). For RNA sequencing, RNA samples were sent to Single Cell Discoveries (Utrecht, the Netherlands) for transcriptome analysis. Samples were multiplexed into a sequencing library and sequenced. Total RNA concentration was measured and normalized to 20 ng/μL. Normalized total RNA (with RNA integrity number (RIN) scores >7) was used for library preparation and sequencing.

To measure apoptosis rate in differentiated C2C12 myocytes, fluorescence‐activated cell sorting (FACS) was used. Cells were stained with 5 μL FITC Annexin‐V and 5 μL propidium ionide staining solution to distinguish early apoptotic cells and late apoptotic cells from living cells.

Data analyses were performed with Prism 8.4.2 (GraphPad Software). For all assessments, experimental groups consisted of at least three biological replicates and all analyses were performed in multiplo. Data are presented as mean ± standard error of the mean. To test for normality of the distribution, a Shapiro–Wilk test was used. Differences between two groups were analysed by an independent samples *t*‐test in case of a normal distribution or a two‐sided Mann–Whitney *U*‐test in case of a skewed distribution. Trends in concentration series were analysed with the ANOVA test followed by a Tukey's multiple comparisons test in case of a normal distribution or a Kruskal–Wallis test followed by Dunn's multiple comparisons test in case of a non‐normal distribution. A *P*‐value of <0.05 was considered statistically significant. In column scatter plots, unless stated otherwise, the median and individual values are presented.

## Results

### Iron deficiency is associated with lower muscle mass in community‐dwelling individuals

**Figure 1:**
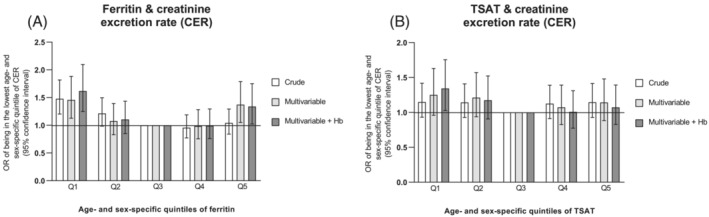
Association between iron status as reflected by age‐ and sex‐specific quintiles of ferritin levels (A) or TSAT (B) and creatinine excretion rate (CER) reflecting muscle mass in community‐dwelling individuals. Odds ratios and corresponding 95% confidence intervals are provided for the risk of being in the lowest age‐ and sex‐specific quintile of 24‐h CER in a crude model (model 1), a multivariable model, adjusted for BMI, eGFR, hs‐CRP, urinary urea excretion, alcohol consumption and smoking status (model 2) and with additional adjustment for haemoglobin (model 3). BMI, body mass index; CER, creatinine excretion rate; eGFR, estimated glomerular filtration rate; hs‐CRP, high sensitive C‐reactive protein; OR, odds ratio; TSAT, transferrin saturation.

We first studied the association between ID and lower muscle mass in a cohort of 5571 individuals (51% men, median age 52 (44–63) years, eGFR 92 ± 17 mL/min/1.73 m^2^, plasma Hb 13.7 ± 1.2 g/dL and plasma ferritin 97 (48–173) μg/L). Further participant characteristics are provided in Table 1. Individuals in the lowest age‐ and sex‐stratified quintile of plasma ferritin level had a significantly higher risk of having low muscle mass, defined as the lowest age‐ and sex‐ specific quintile of CER, compared with the middle quintile (Figure 1). The association remained significant upon adjustment for BMI, eGFR, hs‐CRP, urinary urea excretion, alcohol consumption and smoking status (Model 2, OR 1.46, 95% CI 1.13–1.88, *P* = 0.004), and upon further adjustment for plasma haemoglobin (Model 3, OR 1.62, 95% CI 1.25–2.10, *P* < 0.001). Results were similar in secondary analyses where CER was indexed for length (fully adjusted OR 1.48, 95% CI 1.15–1.92, *P* = 0.003) or length^2^ (fully adjusted OR 1.76, 95% CI 1.36–2.28, *P* < 0.001, Figure S3). Excluding outliers of ferritin also did not materially change the results (Figure S4). In addition, participants in the highest age‐ and sex‐stratified quintile of plasma ferritin level had a higher risk of being in the lowest age‐ and sex‐ specific quintile of CER (fully adjusted OR 1.34, 95% CI 1.03–1.75, *P* = 0.03, Figure 1).

**Table 1:** Participant characteristics

	Age‐ and sex‐specific quintiles of plasma ferritin levels						
	All (*N* = 5571)	Q1 (*N* = 1079)	Q2 (*N* = 1134)	Q3 (*N* = 1104)	Q4 (*N* = 1139)	Q5 (*N* = 1115)	*P*
Ferritin (μg/L)	97 (48–173)	32 (13–51)	76 (29–95)	114 (46–142)	161 (75–204)	278 (183–379)	
Age (years)	52 (44–63)	52 (43–63)	52 (43–63)	52 (43–62)	53 (44–63)	53 (45–64)	0.02
Sex							0.94
Male (*n*, %)	2785 (50)	547 (51)	561 (50)	553 (50)	569 (50)	555 (50)	
Female (*n*, %)	2786 (50)	532 (49)	573 (51)	551 (50)	570 (50)	560 (50)	
Premenopausal (*n*, % of female)	1328 (48)	303 (57)	304 (53)	263 (48)	250 (44)	208 (37)	<0.001
Alcohol intake (units per week)							<0.001
None (*n*, %)	1373 (25)	330 (40)	272 (24)	253 (23)	276 (25)	242 (22)	
±1 (*n*, %)	949 (17)	198 (19)	185 (17)	225 (21)	167 (15)	174 (16)	
2–7 (*n*, %)	1772 (32)	340 (32)	382 (34)	356 (33)	354 (31)	340 (31)	
8–25 (*n*, %)	1206 (22)	178 (17)	242 (22)	223 (20)	279 (25)	284 (26)	
>25 (*n*, %)	224 (4)	23 (2)	40 (4)	37 (3)	51 (5)	73 (7)	
Smoking (*n*, %)	1519 (27)	255 (24)	319 (28)	305 (28)	350 (31)	290 (26)	0.01
BMI (kg/m^2^)	26.6 ± 4.3	25.8 ± 4.2	26.0 ± 4.1	26.4 ± 4.1	26.9 ± 4.2	27.9 ± 4.6	<0.001
Hb (g/dL)	13.7 ± 1.2	13.3 ± 1.4	13.6 ± 1.1	13.8 ± 1.1	13.9 ± 1.1	14.0 ± 1.1	<0.001
Iron (μg/dL)	88.3 ± 31.3	76.5 ± 33.7	87.4 ± 29.2	88.8 ± 29.6	92.2 ± 30.4	95.8 ± 30.2	<0.001
TSAT (%)	25 ± 9	20 ± 10	25 ± 8	25 ± 8	27 ± 9	28 ± 10	<0.001
eGFR (mL/min/1.73 m^2^)	92 ± 17	93 ± 17	92 ± 17	92 ± 17	91 ± 17	91 ± 18	0.07
CRP (mg/L)	1.4 (0.6–3.0)	1.1 (0.5–2.4)	1.1 (0.5–2.6)	1.2 (0.6–2.7)	1.5 (0.7–3.4)	2.0 (0.9–3.8)	<0.001
CER (mmol/24 h)	12.4 ± 3.3	12.1 ± 3.0	12.2 ± 3.2	12.5 ± 3.2	12.5 ± 3.4	12.7 ± 3.5	<0.001
Urea excretion (mmol/24 h)	365 ± 111	357 ± 103	360 ± 109	365 ± 108	368 ± 113	373 ± 118	0.01
Use of antiplatelet drugs (*n*, %)	309 (6)	84 (8)	77 (7)	49 (5)	43 (4)	56 (5)	<0.001
Anticoagulant use (*n*, %)	100 (2)	22 (2)	24 (2)	16 (2)	22 (2)	16 (2)	0.62

BMI, body mass index; Hb, haemoglobin; TSAT, transferrin saturation; eGFR, estimated glomerular filtration rate; CRP, C‐reactive protein; CER, Urinary creatinine excretion.

Participants in the lowest versus the median age‐ and sex‐stratified quintile of plasma TSAT also had a higher risk of being in the lowest age‐ and sex‐ specific quintile of CER after multivariable adjustment (Model 3: OR 1.34, 95% CI 1.03–1.75, *P* = 0.03, Figure 1). Results were similar if CER was indexed for length (fully adjusted OR 1.34, 95% CI 1.02–1.74, *P* = 0.03), and slightly weaker, but with a similar trend for CER indexed for length^2^ (Figure S3). The risk of lower CER for those in the lowest age‐ and sex‐specific quintile of TSAT was attenuated after exclusion of outliers (Figure S4). There was a non‐significant trend suggesting a higher risk of being in the lowest age‐ and sex‐ specific quintile of CER in participants with a higher TSAT (Figure 1), which was not consistent in secondary analyses (Figures S3 and S4).

### Deferoxamine induces iron deficiency and reduces myoblast proliferation but does not affect differentiation to myocytes

**Figure 2:**
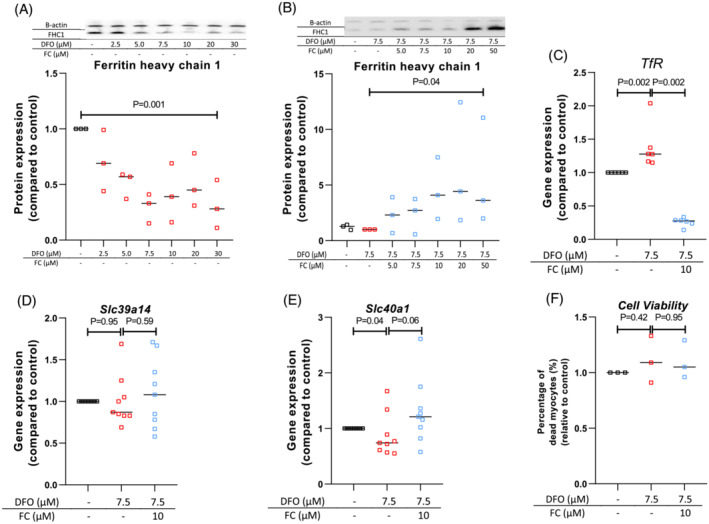
Induction of intracellular ID by DFO is reversed by FC in C2C12 myoblasts and myocytes. (A, B) Protein quantification of Fth in myocytes assessed with Western blot analysis after incubation with or without DFO ± FC for three days. All measurements are normalized for total protein content, for β‐actin content and for untreated controls. Experiments performed in differentiated myocytes are depicted as open squares. Data are based on three separate experiments each. (C–E) Expression of genes encoding proteins for cellular iron uptake: *TfR* (C), *Slc39a14* (D) and *Slc40a1* (E) assessed with qPCR techniques in myocytes, after incubation with or without DFO and FC for one day. Measurements are normalized for housekeeping gene expression and for untreated controls. Data are based on six to nine separate experiments each. (F) Percentage of dead myocytes assessed with flow cytometry techniques. Data are based on three separate experiments. Experiments performed without DFO or FC are depicted as black symbols, experiments with DFO as red symbols and experiments with DFO and FC as blue symbols.

Incubation of C2C12 skeletal myoblasts and myocytes with DFO for three days resulted in dose‐dependently reduced concentrations of Fth (Figure 2A, Figure S5A). Co‐incubation of myoblasts and myocytes with increasing concentrations of FC in addition to 7.5 μM DFO dose‐dependently restored concentrations of Fth (Figure 2B, Figure S5B). Gene expression of Transferrin receptor (*TfR*), involved in cellular uptake of transferrin‐bound iron, increased with 28% by 7.5 μM DFO treatment of myocytes for one day (*P* = 0.002) and decreased by 78% upon co‐treatment with 10 μM FC (*P* = 0.002, Figure 2C). Expression of *Slc39114*, encoding the iron uptake marker ZIP14, was not affected by treatment with DFO or FC (Figure 2D). Expression of *Slc40a1*, encoding ferroportin, a cellular iron export protein, was reduced after DFO treatment (*P* = 0.04, Figure 2E), which was reversed by co‐treatment with FC (Figure 2E). DFO or FC did not affect cell viability (Figure 2F).

**Figure 3:**
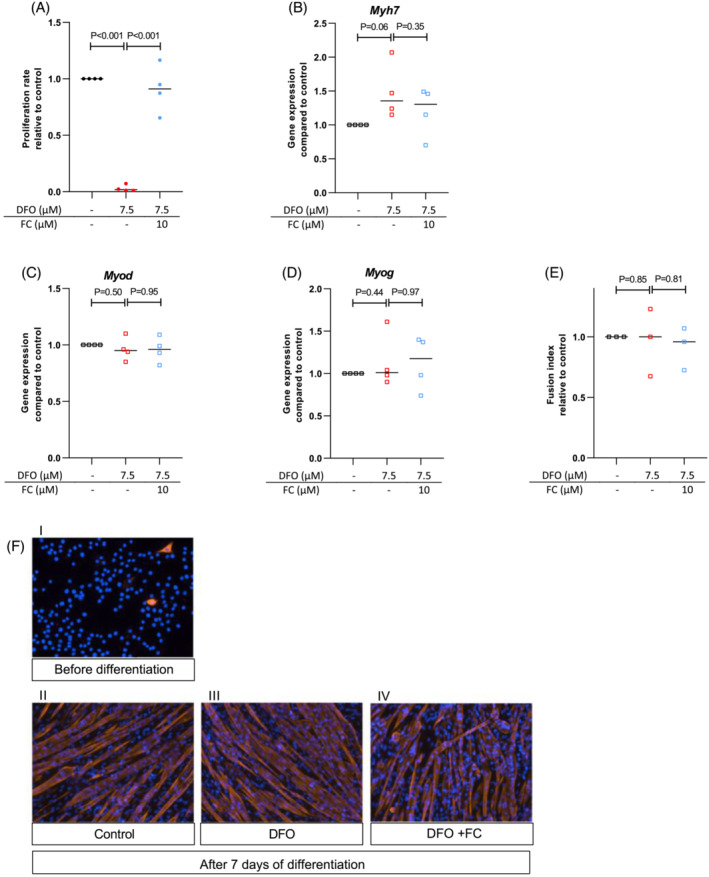
Treatment of C2C12 myoblasts with DFO leads to impaired proliferation rate while additional treatment with FC rescues proliferation rate. Treatment with DFO with or without FC does not affect C2C12 myoblast differentiation rate. Proliferation rate was assessed with a BrdU cell proliferation ELISA assay. (A) Proliferation rate with or without DFO and/or FC. Data are based on four separate experiments each. (B–D) Expression of genes involved in myoblast differentiation into myocytes using qPCR techniques, after incubation with or without DFO and FC for two days: Myh7 (B), Myod (C) and Myog (D). Measurements are normalized for housekeeping gene expression and for untreated controls. Data are based on three separate experiments each. (E) Differentiation rate assessed using staining techniques after incubation with or without DFO and FC for two days. Data are based on three experiments each. (F) Representative images of anti‐Myh3 staining of C2C12 cells before (I) or after (II, Blanco; III, with DFO; IV, with DFO and FC) incubation with differentiation medium. Experiments performed without DFO or FC are depicted as black symbols, experiments with DFO as red symbols and experiments with DFO and FC as blue symbols.

In C2C12 myoblasts, DFO dose‐dependently reduced the proliferation rate (*P*‐trend <0.001, Figure S6). Co‐incubation with 10 μM FC fully restored proliferation rate (*P* < 0.001, Figure 3A). DFO treatment with or without FC during differentiation of myocytes did not affect expression of differentiation markers *Myh7* (encoding myosin heavy chain), *Myod* (encoding myoblast determination protein 1) and *Myog* (encoding myogenin) (Figures 3B–D) or the fusion index (Figure 3E).

### Iron deficiency leads to mitochondrial dysfunction and reduces myoglobin content in skeletal muscle cells

**Figure 4:**
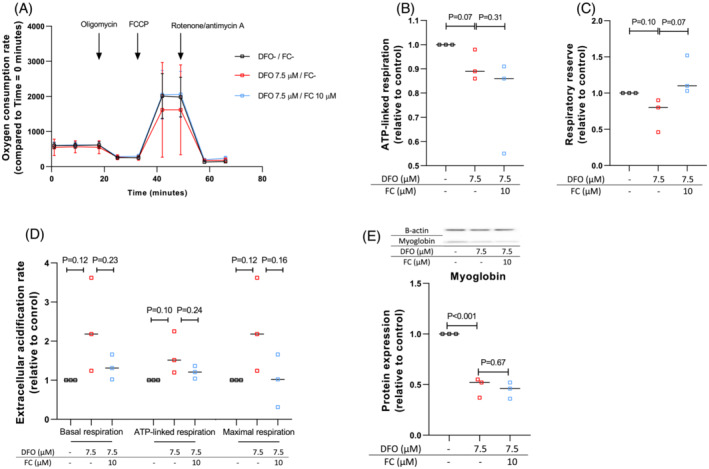
Induction of ID affects mitochondrial function and leads to reduced myoglobin content of C2C12 myocytes. *(*A) Oxygen consumption rate of C2C12 myocytes treated with or without DFO and FC during a mitochondrial stress test. (B) Effects of DFO and FC on ATP‐linked respiration. (C) Effects of DFO and FC on respiratory reserve. (D) Effects of DFO and FC on extracellular acidification rate during basal respiration, ATP‐linked respiration and maximal respiration. Data are based on three separate experiments each. Experiments were performed in multiplo: 7–32 technical replicates were used per experiment. The medians of all technical replicates per experiment are depicted. (E) Protein quantification of myoglobin in myocytes treated with or without DFO and/or FC with Western blot techniques. Measurements are normalized for total protein content, for β‐actin content and for untreated control. Data are based on three separate experiments each. Experiments performed without DFO or FC are depicted as black symbols, experiments with DFO as red symbols and experiments with DFO and FC as blue symbols.

Basal OCR was slightly and non‐significantly lower upon incubation with 7.5 μM DFO (Figure 4A). During a mitochondrial stress test, DFO treatment resulted in a small and borderline significant decline (−9%, *P* = 0.07) in ATP‐synthase linked respiration reflected by the difference between basal respiration rate and remaining respiration rate after oligomycin injection (OCR_basal_ − OCR_oligomycin_), which was not restored by co‐treatment with 10 μM FC (Figure 4B). Subsequently, FCCP was injected to assess respiratory reserve, reflected by the increase in oxygen consumption rate relative to baseline (OCR_FCCP_ − OCR_basal_). Myocytes treated with DFO tended to have a lower maximal mitochondrial capacity, compared with untreated myocytes (−28%, *P* = 0.10, Figure 4C). Additional treatment with FC restored respiratory reserve. ECAR during basal respiration, ATP‐linked respiration and maximal respiration, reflecting lactate metabolism, increased after addition of DFO and decreased after additional treatment with FC, although these differences did not reach statistical significance (Figure 4D).

We next investigated the effects of ID on oxygen storage and mitochondrial function in differentiated C2C12 myocytes. ID induction reduced myoglobin concentration in C2C12 myocytes (−52%, *P* < 0.001); iron repletion with FC did not restore myoglobin levels (Figure 4E).

### Iron deficiency induces markers of myocyte atrophy and protein degradation

**Figure 5:**
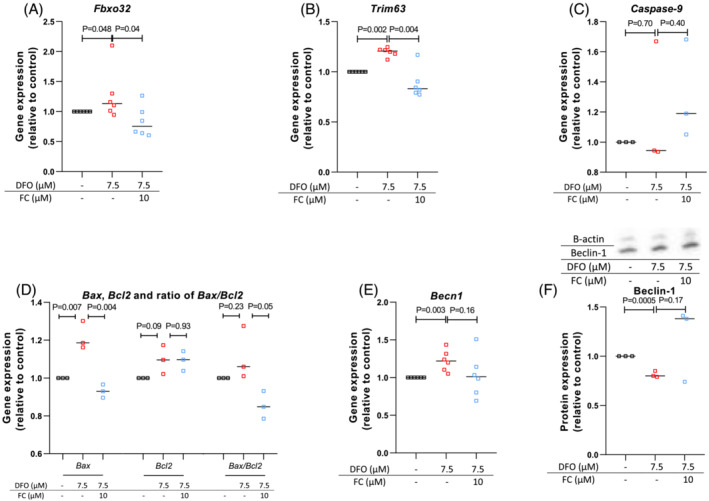
Treatment with 7.5 μM deferoxamine (DFO) induces early cell death. (A–E) Expression status of different genes assessed with qPCR techniques. Measurements are normalized for housekeeping gene expression and for untreated control. Data are based on six separate experiments each for Fbxo32, Becn1 and Trim63 and three experiments each for *Bax*, *Bcl2* and *caspase‐9*. (F) Protein quantification of beclin‐1 in myocytes treated with or without DFO and/or FC with Western blot techniques. Measurements are normalized for total protein content, for β‐actin content and for untreated control. Data are based on three separate experiments each. Experiments performed without DFO or FC are depicted as black symbols, experiments with DFO as red symbols and experiments with DFO and FC as blue symbols.

Addition of DFO to the culture medium for one day significantly increased gene expression of the *Fbxo32*, encoding the protein atrogin‐1 (+27%, *P* = 0.048), and *Trim63*, encoding MuRF‐1 (+20%, *P* = 0.002), both markers of protein degradation and muscle atrophy. Additional treatment with FC significantly decreased expression of *Fbxo32* (−31%, *P* = 0.04) and *Trim63* (−26%, *P* = 0.004, Figure 5A,B). Treatment with DFO also significantly induced expression of *Becn1*, which encodes beclin‐1, a protein involved in protein degradation (+22%, *P* = 0.003). There was a trend towards reduced *Becn1* expression after additional treatment with FC (Figure 5E). Upon protein quantification, beclin‐1 was decreased after treatment with DFO (−19%, *P* = 0.001, Figure 5F). Treatment with DFO for one day significantly induced expression of the apoptosis gene *Bax* (+25%, *P* = 0.007) and tended to increase *Bax/Bcl2* ratio (+20%), and to increase the percentage of late apoptotic cells (Figure 5D). Co‐treatment with FC significantly decreased *Bax* expression (−21%, *P* = 0.004) and *Bax/Bcl2* ratio (−21%, *P* = 0.047), while it did not significantly alter the total percentage of early apoptotic or necrotic myocytes (Figure S7). Caspase‐9 expression was not modified by DFO or FC (Figure 5C).

### Iron deficiency affects key pathways of energy and nucleotide metabolism

**Figure 6:**
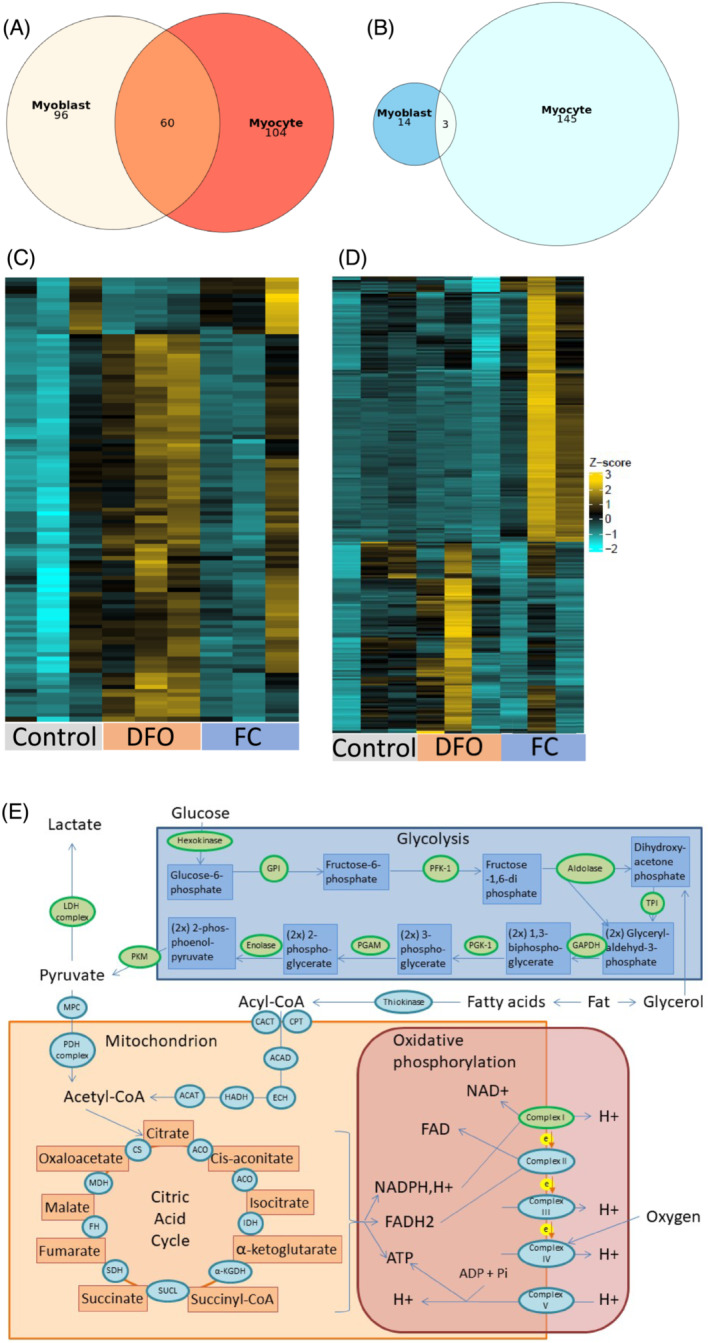
Treatment with 7.5 μM deferoxamine (DFO) leads to altered gene expression in myoblasts and myocytes. (A, B) Scaled Venn diagrams of differentially expressed genes (DEGs): Genes of which the expression is increased after treatment with DFO and reduced after additional treatment with FC (A) or genes of which the expression is reduced after treatment with DFO and increased after additional treatment with FC (B). (C, D) Heatmap of DEGs with or without treatment with DFO and/or FC in myoblasts (C) and myocytes (D), illustrating clear differences in gene expression patterns between the groups. (E) Schematic overview of cellular carbohydrate and fat energy metabolism. The key enzymes involved in these processes are depicted as circles. Enzymes of which the gene expression is not affected by DFO are depicted in blue. Enzymes of which the gene expression is increased after treatment with DFO are depicted in green. None of the enzymes were down‐regulated after treatment with DFO. ACAD, acyl‐coenzyme a dehydrogenase; ACAT, acetyl‐ coenzyme a acetyltransferase; ACO, aconitase; ADP, adenosine diphosphate; ATP, adenosine triphosphate; CACT, carnitine‐acyl carnitine translocase; CPT, carnitine palmitoyltransferase; CS, citrate synthase; DFO, deferoxamine; ECH, enoyl‐coenzyme a hydratase; FAD, flavin adenine dinucleotide; HADH, hydroxyacyl‐coenzyme a dehydrogenase; FC, ferric citrate; FH, fumarate hydratase; GAPDH, glycerylaldehyde 3‐phosphate dehydrogenase; GPI, glucose‐6‐phosphate isomerase; HADH, hydroxyacyl‐coenzyme a dehydrogenase; IDH, isocitrate dehydrogenase; α‐KGDH, α‐ketoglutarate dehydrogenase; LDH, lactate dehydrogenase; MDH, malate dehydrogenase; MPC, mitochondrial pyruvate carrier; NAD+, nicotinamide adenine dinucleotide; NADPH, nicotinamide adenine dinucleotide phosphate; PDH, pyruvate dehydrogenase; PFK‐1, phosphofructokinase 1; PGAM, phosphoglycerate mutase; PGK‐1, phosphoglycerate kinase; PKM, pyruvate kinase; SDH, succinate dehydrogenase; SUCL, succinyl‐coenzyme A‐synthetase; TPI, triose phosphate isomerase.

To explore potential mechanisms explaining impaired proliferation, mitochondrial respiration and increased apoptosis, we profiled transcriptomic changes in C2C12 myoblasts and myocytes after treatment with DFO for one day with or without 10 μM FC. RNA sequencing revealed 110 differentially‐expressed genes (DEGs) in myoblasts compared with 270 DEGs in myocytes; 65 DEGs overlapped between the cell types. In myoblasts, expression of 96 genes was significantly up‐regulated after treatment with DFO and restored after co‐treatment with FC, while 14 genes showed the opposite pattern. In myocytes, 104 genes were up‐regulated after treatment with DFO and restored after additional treatment with FC, and 145 genes showed the opposite pattern (Figure 6A–D). Table 2 shows genes with most significant differences upon DFO treatment with or without FC.

**Table 2:** Top five genes with most significant change after ID induction and that changed in the opposite direction after co‐treatment with FC

				Change after ID induction		Change after co‐treatment with FC	
	Gene	Protein	Function	Fold change	*P*‐value	Fold change	*P*‐value
Up‐regulated by DFO, down‐regulated by FC, myoblasts							
1	*Bnip3*	Bcl2 interacting protein 3	Induction of apoptosis	4.6	1.84E‐66	0.20	6.18E‐76
2	*Fam162a*	Human growth and transformation‐dependent protein	Activation of apoptosis in the context of HIF‐1α stabilization	3.2	1.86E‐42	0.30	9.09E‐46
3	*Higd1a*	HIG1 hypoxia inducible domain family 1A	Promotor of mitochondrial homeostasis and cell survival in the context of hypoxia	3.4	3.71E‐28	0.28	5.89E‐32
4	*Pdk1*	Pyruvate dehydrogenase kinase 1	Inhibition of pyruvate conversion to Acetyl‐CoA, main substrate of mitochondrial tricarboxylic acid cycle	3.4	6.59E‐25	0.27	4.77E‐30
5	*Pgm2*	Phosphoglucomutase 2	Glycogenolysis/glycogenesis	2.6	1.35E‐23	0.38	8.40E‐25
Down‐regulated by DFO, up‐regulated by FC, myoblasts							
1	*Shmt2*	Serine hydroxymethyl‐transferase 2	Folic acid cycle	0.49	3.83E‐14	2.0	1.49E‐13
2	*Gsta4*	Glutathione S‐transferase alpha 4	Detoxification of products of oxidative stress	0.61	4.83E‐08	1.9	1.41E‐12
3	*Mthfd2*	Methylenetetrahydro‐folate dehydrogenase (NADP+)2	Folic acid cycle	0.51	7.33E‐08	1.9	8.93E‐08
4	*Phgdh*	Phosphoglycerate dehydrogenase	Serine biosynthesis	0.60	1.38E‐06	1.6	5.36E‐06
5	*Gnl3*	G protein nucleolar 3	Cell cycle, proliferation	0.60	8.27E‐06	1.6	5.65E‐05
Up‐regulated by DFO, down‐regulated by FC, myocytes							
1	*Car9*	Carbonic anhydrase	Conversion of CO2 and H2O into HCO3‐ and H+	3.6	8.06E‐21	0.07	8.24E‐107
2	*Egln1*	Egl‐9 family hypoxia inducible factor 1	Activation of hypoxic‐response gene transcription	2.2	1.11E‐08	0.37	3.03E‐33
3	*Ier3*	Immediate early response 3	Marker of cellular stress, cell cycle control, apoptosis	2.7	7.89E‐09	0.29	8.98E‐35
4	*Ero1L*	ERO1‐like protein alpha	Activation of apoptosis in the context of HIF‐1α stabilization	2.8	1.82E‐08	0.26	1.69E‐37
5	*Fam162a*	Human growth and transformation‐dependent protein	Induction of apoptosis in the context of hypoxia	2.0	9.16E‐03	0.24	1.86E‐63
Down‐regulated by DFO, up‐regulated by FC, myocytes							
1	*Nov*	Nephroblastoma overexpressed	Inhibitor of osteoblast function (in osteoblasts)	0.57	7.87E‐09	2.3	5.87E‐19
2	*Abca1*	ATP binding cassette subfamily A member 1	Transmembrane transport of lipids	0.46	1.74E‐06	2.1	7.77E‐06
3	*Slc1a3*	Solute carrier family 1 member 3	Amino acid transport	0.61	3.30E‐06	1.6	5.26E‐06
4	*Aldh3a1*	Aldehyde dehydrogenase 3 family member A1	Detoxification	0.61	1.55E‐05	2.0	3.12E‐09
5	*Ccnd1*	Cyclin D1	Cell cycle	0.66	1.42E‐04	1.5	1.55E‐04

Next, we assessed whether alterations in genes related to energy metabolism could explain reduced mitochondrial function in iron‐deficient myocytes. Expression of genes encoding all major enzymes involved in the anaerobic steps of glucose metabolism, glycolysis and lactate production was up‐regulated in myocytes after treatment with DFO (Figure 6E). On the contrary, apart from two subunits of complex I of the electron transport chain, none of the genes encoding enzymes involved in the mitochondrial steps of energy metabolism and in fatty acid oxidation (both oxygen‐dependent) were affected (Figure 6E).

Although RNA sequencing analysis did not confirm significantly altered expression of *Fbxo32*, *Trim63*, *Becn1* or *Bax*, we did observe up‐regulated expression of pro‐apoptotic genes *Ddit4*, *Bnip3*, *Bnip3L*, *Fam162a* and *Ero1L* in iron‐deficient myoblasts and myocytes. Pathway enrichment analysis showed that in myoblasts, ID most strongly affected energy metabolism, nucleotide metabolism and hypoxic responses (Figure S8a). In differentiated myocytes, nucleotide metabolism, cell cycle regulation and hypoxic responses were most affected (Figure S8b). Computational analysis of literature‐based putative transcription factors showed that transcription factors that best predicted clusters of identified DEGs after ID induction were *Hif1A*, encoding hypoxia inducible factor subunit 1‐alpha, in myoblasts and *Foxm1*, a gene involved in cell proliferation, in myocytes (Table S2).

## Discussion

To our knowledge, this is the first study to reveal a relation between ID and reduced muscle mass in the general population, independent of haemoglobin levels and potential confounders. In cultured skeletal muscle cells, we found that ID strongly reduces proliferation in myoblasts and adversely impacts pathways related to energy and nucleotide metabolism in myocytes, potentially explaining our observations in humans.

Our findings in humans are in line with previous studies, mostly in disease populations. In patients with chronic heart failure, ID was associated with impaired exercise tolerance and muscle strength,^9^, ^10^, ^19^, ^20^, ^21^ while ID correction improved exercise capacity^22^, ^23^, ^24^, ^25^ and cardiac function.^11^, ^26^ Also, in the elderly, lower iron status has been related with lower muscle strength.^27^ Intravenous iron treatment of iron‐deficient cancer patients enhanced muscle strength.^28^ Finally, ID was associated with less recovery of muscle strength after stroke.^29^ Other studies report a detrimental effect of ID on endurance. In women, a lower TSAT was correlated with worse aerobic fitness, but not with anaerobic capacity^30^ while correction of non‐anaemic ID improved peak VO_2_ during exercise.^31^ Chronic heart failure patients with ID displayed more pronounced phosphocreatine depletion in calf muscle after exercise and a higher muscular acidification rate, or a rapid switch to anaerobic glycolysis,^10^ while intravenous iron reduced phosphocreatine depletion.^11^

Not only low, but also higher ferritin levels were linked to lower muscle mass in our study. A negative correlation between ferritin and muscle mass has been previously observed in the NHANES cohort.^32^ Iron overload triggers the Fenton reaction, inducing the formation of oxygen radicals, which may have detrimental effects on skeletal muscle.^33^, ^34^ Furthermore, ferritin is an acute‐phase protein and high levels may occur in the context of inflammation, although the relation between ferritin and muscle mass remained significant after adjustment for CRP in our study.

Iron is a main component of haemoglobin, and thereby plays an important role in transport of oxygen to all tissues, including skeletal muscle.^35^ Furthermore, iron is involved in nucleotide synthesis and cellular energy metabolism, and may therefore be crucial for cell proliferation and function.^35^ Finally, iron is important for biosynthesis of testosterone^36^, ^37^ which promotes muscle mass.

To further study the effects of ID on skeletal muscle function, we used C2C12 cells, a well‐documented model of immortalized mouse myoblasts that can differentiate into skeletal myocytes. ID was induced using the iron‐chelator DFO. A reduction in intracellular Fth content confirmed that DFO captured iron from C2C12 myoblasts and differentiated myocytes. Addition of 7.5 μM DFO caused a > 50% reduction in Fth content, and therefore this concentration was used for further experiments. Co‐treatment with the iron compound FC prevented the increase in intracellular Fth in myoblasts and myocytes. The expression of TfR, a marker of cellular ID, was increased by DFO, while expression of Slc40a1, a marker of iron abundance, was decreased (Figure 2C–E). Co‐treatment with FC prevented these changes, and therefore we considered these conditions to be representative of iron depletion and repletion, respectively, in skeletal myoblasts and myocytes.

DFO significantly impaired myoblast proliferation with a clear dose‐effect response. To confirm that the reduction in proliferation rate was attributed to ID, rather than the chelation of other metal ions or direct toxic effects of DFO, iron availability was restored by co‐treatment with FC, which reversed the effects of DFO (Figure 3A). There are several possible explanations for this observation. First, proliferation could be reduced due to ID‐induced adverse effects on energy metabolism. Furthermore, the observation from RNA sequencing and gene ontology that nucleotide synthesis is one of the main processes affected by ID also suggests direct effects on DNA replication. In myoblasts, two of the five genes that were most significantly suppressed were involved in the folic acid cycle, which is crucial for DNA stabilization and replication. DFO did not affect myocyte differentiation.

Subsequently, we assessed the effects of ID on energy metabolism. Although oxygen consumption during basal cellular respiration was not affected by ID, there was a non‐significant trend suggesting a lower maximal mitochondrial reserve. Also, the acidification rate was higher after ID induction, although this was also non‐significant. Previous studies showed a significant reduction in basal respiratory consumption rate as well as in maximal oxygen consumption in human cardiomyocytes treated with DFO^5^ or in C2C12 skeletal muscle cells treated with deferiprone, another iron chelator.^13^ Using RNA sequencing analysis, we found that expression of most genes encoding proteins involved in glycolysis and lactate production, the main processes of anaerobic energy metabolism, was up‐regulated after ID induction. In contrast, expression of genes involved in aerobic processes such as fatty acid oxidation, the citric acid cycle and oxidative phosphorylation, was not affected, apart from two subunits of complex I. This finding is in line with a previous study in cardiomyocytes^5^ and suggests that during ID, there is a malfunction of the oxidative energy metabolism and a compensatory shift towards the glycolytic anaerobic type of chemical energy production. Activity of the citric acid cycle may be hampered by a lower function of aconitase, which is known to be iron‐dependent,^38^ while oxidative phosphorylation may be impaired because the five complexes driving oxidative phosphorylation contain iron in the form of iron–sulfur clusters.^39^ Moreover, their function requires oxygen, which may be insufficiently available because of a lower content of myoglobin, as also observed in the current study and, previously, in mice.^14^ Our findings are in line with previous observations in iron‐deficient rodents in whom complex I‐IV activity was reduced in muscle.^14^, ^40^ Although glycolysis is usually sufficient for fast and powerful muscle contractions, oxidative metabolism of fatty acids and carbohydrates is required for sustained skeletal muscle use. A shift from aerobic towards anaerobic energy metabolism is a common hallmark of chronic diseases such as diabetes, heart failure or chronic obstructive pulmonary disease.^41^ In patients with chronic heart failure, correction of ID improved skeletal muscle energy metabolism.^11^

We also found that ID induced expression of two genes that are important for cellular atrophy and protein degradation, *Fbxo32* and *Trim63*, in line with a previous study.^42^ Our results regarding the effects of ID on apoptosis were not equivocal: expression of both *Bax* and *Bcl2* was slightly but non‐significantly increased and the expression of caspase‐9 was not altered. However, the expression of *Bnip3*, a pro‐apoptotic gene encoding *Bcl2* interacting protein 3, the highly similar *Bnip3L*, and *Ddit4*, encoding a marker of hypoxia and DNA damage, was increased in iron‐deficient myoblasts. Also, expression of *Fam162a* and E*ro*1L, involved in hypoxia‐induced apoptosis, was increased in iron‐deficient myoblasts and myocytes. We did not find a change in percentages of dead, early apoptotic or total apoptotic cells, although there was a minor but significant increase in the percentage of late apoptotic cells. In addition, we found ID‐induced up‐regulation of *Becn1*, encoding beclin‐1 which is the most important inducer of autophagy, suggesting that myocytes promote autophagy to recycle iron when its availability is scarce. However, protein expression of beclin‐1 was reduced, not increased. This may be explained by posttranscriptional suppression of beclin‐1 by apoptotic processes, known to regulate autophagy.^43^ Potentially, induction of apoptosis could contribute to loss of muscle mass in patients with ID.

Computational prediction of putative transcription factors analysis revealed that hypoxia inducible factor subunit 1‐alpha (HIF1α) is the transcription factor involved in the expression of the largest cluster of DEGs related to ID. HIF1 is stabilized in response to hypoxia, promoting erythropoiesis and iron uptake from the gut, but may also be up‐regulated by ID, independent of oxygen availability.^44^ HIF1 may drive cellular compensatory mechanisms, such as inhibition of fatty acid oxidation and promotion of glycolysis and mitochondrial autophagy in response to skeletal muscle hypoxia.^45^ Possibly, up‐regulation or stabilization of HIF1α is the central effect of ID in skeletal muscle, explaining our other observations of altered energy metabolism and induction of cell‐degradation. This hypothesis is partially supported by the observations of Leermakers et al, who reported that the increased expression of pro‐apoptotic *Bnip3* was HIF1A‐dependent.^13^

Our study adds important new insights in the effects of ID on skeletal muscle. Its main strength is the availability of data from a large and extensively phenotyped cohort of community‐dwelling individuals, combined with results of *in vitro* studies to investigate underlying mechanisms. Our study also has a number of limitations. First, we were not able to assess the relationship of ID with skeletal muscle strength in humans or with contractile strength in myocytes. Second, we did not assess morphological effects of ID on myocytes and therefore we could only provide circumstantial evidence that ID may induce atrophy and programmed cell death. Third, because we used a cell model, we could not assess any effects of ID on tissue vascularization, which might be increased by the HIF1a signalling cascade. Finally, the *in vitro* setting in C2C12 cells and the use of DFO to induce ID might not accurately reflect the impact of ID *in vivo*, and therefore the observations in C2C12 cells cannot be directly translated to the human *in vivo* setting. Nevertheless, our findings provide mechanistic insights that are supportive of the observations in humans.

In conclusion, ID was consistently associated with lower muscle mass in community‐dwelling individuals, independent of haemoglobin and other factors. In skeletal muscle cells, ID reduced proliferation and resulted in increased expression of genes related to atrophy, apoptosis and autophagy, while inducing a switch from aerobic to anaerobic energy metabolism. Future studies should address whether correction of ID improves muscle mass.

## Conflict of interest

JSJ Vinke has received speaker fees from Vifor Pharma (paid to employer). MF Eisenga has declared receiving consultant fees from Vifor Pharma; serving on the Advisory Board for Cablon Medical; and receiving speaker's bureaus from Vifor Pharma, Cablon Medical, and Astellas (all to employer). P vd Meer received grant support and/or consultancy fees from: Novartis, Pharma Nord, Pfizer, Ionis, Astra Zeneca, Vifor Pharma, Pharmacosmos, BridgeBio, NovoNordisk. MH de Borst has consultancy agreements with Amgen, Astellas, Astra Zeneca, Bayer, Kyowa Kirin, Vifor Fresenius Medical Care Renal Pharma, and Sanofi Genzyme, and received grant support from Sanofi Genzyme and Vifor Pharma (all to employer). AR Gorter, WA Dam, J vd Born, SJL Bakker and MF Hoes declared that they have no conflict of interest.

## Funding

This work has been supported by the Dutch Kidney Foundation (grant no 17OKG18).

## Supporting information


**Table S1.** Mouse primers for RT‐PCR.
**Table S2.** Top ten transcription factors known, based on literature, to be involved in expression of the largest number of differentially expressed genes after induction of iron deficiency.

**Figure S1.** Flowchart of inclusion.

**Figure S2.** Differentiation of C2C12 myoblasts to myocytes after addition of differentiation medium. Gene expression of differentiation markers Myh7 (A), Myod (B) and Myog (C) before and after seven days of culturing with differentiation medium. Measurements are normalized for housekeeping gene expression and for untreated controls. (D) Fusion index before and after seven days of culturing with differentiation medium. Data are based on three separate experiments each.

**Figure S3.** Association between iron status as reflected by ferritin levels (A, C) or TSAT (B, D) and CER indexed for length (A, B) or CER indexed for length squared (C, D) in community‐dwelling individuals. Odds ratios and corresponding 95% confidence intervals are provided for the risk of being in the lowest age‐ and sex‐specific quintile of 24‐hour CER in a crude model (Model 1), a multivariable model, adjusted for BMI, eGFR, hs‐CRP, urinary urea excretion, alcohol consumption and smoking status (Model 2) and with additional adjustment for haemoglobin (Model 3).
Abbreviations: CER, creatinine excretion rate; TSAT, transferrin saturation; eGFR, estimated glomerular filtration rate; BMI, body mass index; hs‐CRP, high sensitive C‐reactive protein; OR, odds ratio.

**Figure S4.** Association between iron status as reflected by ferritin levels (A) or TSAT (B) and CER in community‐dwelling individuals, after excluding the 5% most extreme outliers of ferritin (A) or TSAT (B). Odds ratios and corresponding 95% confidence intervals are provided for the risk of being in the lowest age‐ and sex‐specific quintile of 24‐hour CER in a crude model (Model 1), a multivariable model, adjusted for BMI, eGFR, hs‐CRP, urinary urea excretion, alcohol consumption and smoking status (Model 2) and with additional adjustment for haemoglobin (Model 3).
Abbreviations: CER, creatinine excretion rate; TSAT, transferrin saturation; eGFR, estimated glomerular filtration rate; BMI, body mass index; hs‐CRP, high sensitive C‐reactive protein; OR, odds ratio.

**Figure S5.** Induction of intracellular ID by DFO is reversed by FC in C2C12 myoblasts. Protein quantification of Fth in myoblasts assessed with Western Blot analysis after incubation with or without DFO without (A) or with (B) FC for three days. All measurements are normalized for total protein content, for β‐actin content and for untreated controls. Experiments performed in myoblasts are depicted as closed circles. Data are based on three separate experiments each. Experiments performed without DFO or FC are depicted as black symbols, experiments with DFO as red symbols and experiments with DFO and FC as blue symbols.

**Figure S6.** Treatment of C2C12 myoblasts with DFO leads to impaired proliferation rate. Proliferation rate was assessed with a BrdU cell proliferation ELISA assay under increasing concentrations of DFO. Data are based on three separate experiments each. Experiments performed without DFO or FC are depicted as black symbols, experiments with DFO as red symbols and experiments with DFO and FC as blue symbols.

**Figure S7.** Treatment with 7.5 μM deferoxamine (DFO) may induce apoptosis. Percentage of apoptotic or necrotic cells assessed with flow cytometry techniques. Data are based on three separate experiments each. Experiments performed without DFO or FC are depicted as black symbols, experiments with DFO as red symbols and experiments with DFO and FC as blue symbols.

**Figure S8.** Revigo TreeMap of Gene Ontology (GO) terms reflecting biological processes in which the differentially expressed genes under deferoxamine treatment in myoblasts (A) and myocytes (B) are involved. Each rectangle represents a GO term and related terms are combined into clusters with the same colour. The size of the rectangles represents the frequency of the GO term related to the differentially expressed genes as well as the P‐value.
