# Supplementary material for: Iron deficiency is related to lower muscle mass in community‐dwelling individuals and impairs myoblast proliferation
Source: J Cachexia Sarcopenia Muscle. 2023 Jun 30;14(4):1865–79. doi: 10.1002/jcsm.13277 (PMC10401536; doi:10.1002/jcsm.13277)
Supplement: Supplementary file 1 — Table S1. Mouse primers for RT‐PCR. Table S2. Top ten transcription factors known, based on literature, to be involved in expression of the largest number of differentially expressed genes after induction of iron deficiency. [file JCSM-14-1865-s008.docx]

**Supplemental Material**

**List of contents**

1. Extensive methods: page 1

1.1 Human studies: page 1

1.2 Cell culture studies page 3

2. Supplemental tables page 12

3. Supplemental figures page 13

1. **Extensive methods**
   1. ***Human studies***

***Study Population***

We used cross-sectional data from the Prevention of Renal and Vascular End-stage Disease (PREVEND) study, a prospective, population-based cohort of Dutch community-dwelling individuals aged 25 to 75 years. In this study, 8592 participants were enrolled at baseline. For the current analysis, we analyzed data from the second survey (2001-2003). We excluded participants with missing data on urinary creatinine excretion rate (CER) or ferritin (Supplemental Figure 1). To limit potential 24-hour urine collection errors, we excluded participants at the lowest or highest 2.5% of difference between expected and measured 24-hour urine volume[S1]. Eventually, 5571 participants remained available for analysis. The study was approved by the local ethics committee and complied with the principles of the Declaration of Helsinki. Written informed consent was obtained from all participants before enrollment.

***Assays and definitions***

Fasting blood samples were collected in the morning. Plasma creatinine was assessed using an enzymatic assay on a Roche Modular Analyzer (Roche Diagnostics, Mannheim, Germany). Glomerular filtration rate (eGFR) was estimated using the Chronic Kidney Disease Epidemiology Collaboration (CKD-EPI) creatinine and cystatin-C-based equation[S2]. All participants were asked to collect two consecutive 24-h urine samples before their visit to the clinic. The average CER of the two samples, calculated from total urine volume and creatinine concentrations, was used as a parameter for muscle mass. The average urinary urea excretion of the two samples was used as a parameter of protein intake and nutritional status. The expected 24-hour urine volume was calculated with the following equation: 24-hour urine volume = (plasma creatinine x creatinine clearance) / urinary creatinine, and creatinine clearance was calculated by the Cockcroft-Gault formula[S3]. Hemoglobin level was measured using a Coulter Counter STKS sum (Coulter Corporation, Miami, FL). Plasma iron was measured using a colorimetric assay, ferritin was measured using immunoassay and transferrin was measured using an immunoturbidimetric assay (Roche Diagnostics, Mannheim, Germany). Transferrin saturation (TSAT, %) was calculated as 100 x plasma iron (µmol/L) ÷ 25 x transferrin (g/L). Plasma and urine creatinine were performed using an isotope dilution mass spectrometry traceable enzymatic method (Roche Diagnostics, Mannheim, Germany). Anemia was defined as a hemoglobin level (Hb) <12 g/dL for women and <13 g/dL for men, according to the WHO definition. Body Mass Index (BMI) was calculated as weight in kilograms divided by length in meters squared.

***Statistical analyses***

IBM SPSS Statistics version 23.0 (SPSS Inc., Chicago, IL) was used to analyze the data. Normally distributed data are presented as mean ± standard deviation (SD) while data with a skewed distribution are presented as median (IQR). Categorical data are expressed as number (percentage). In all analyses, a *P* value of ≤0.05 was considered significant.

CER, plasma ferritin levels and TSAT were divided into quintiles after stratification for age (below or above the median age of 52 years) and sex. Between-group differences in participants in the different age- and sex-specific quintiles of plasma ferritin level were assessed using the ANOVA test for normally distributed variables, the Kruskal Wallis Test for variables with a skewed distribution, and the Chi square test for categorical data.

To optimally adjust for the confounding effect of age and sex, on which ferritin levels as well as muscle mass are strongly dependent, we used logistic regression analyses to assess associations between plasma ferritin levels or TSAT and CER. The odds ratio of being in the lowest quintile of age- and sex-stratified CER was compared between participants across quintiles of age- and sex-stratified ferritin and TSAT levels, using logistic regression (model 1). Upon multivariable analysis (model 2), we adjusted for estimated glomerular filtration rate (eGFR), body mass index (BMI), high-sensitive C-reactive protein (hs-CRP), 24-hour urinary urea excretion (reflecting protein intake), alcohol consumption and smoking status. In model 3, we additionally adjusted for plasma hemoglobin (Hb).

In sensitivity analyses, we substituted CER for length-indexed CER[S4] and length^2-indexed CER[S5], given that muscle mass depends on body size. Furthermore, we repeated all analyses after exclusion of the 5% most extreme ferritin or TSAT values by calculating absolute differences from the median.

- 1. ***Cell culture studies***

***Cell proliferation***

Proliferation rate of C2C12 myoblasts was determined using a colorimetric 5-bromo-2′-deoxy-uridine (BrdU) cell proliferation ELISA kit (Abcam, Cat. No. ab26556). C2C12 myoblasts were plated with a seeding density of 2625 cells/cm^2^ (875 cells/well) on a 96-well plate. The next day, DFO with or without FC was applied to the cells in culture medium, and left to incubate for 72 hours. 21 hours before the start of the immunodetection, BrdU reagent was added to the cells. After BrdU incorporation, cells were first fixated, permeabilized and DNA was fragmentized by incubating the cells with fixing solution for 30 minutes at room temperature. This was followed by three wash cycles using washing buffer. The cells were incubated with primary anti-BrdU monoclonal antibody for one hour at room temperature. After three more washing cycles, secondary goat anti-mouse IgG HRP-conjugate antibody was added to the cells and incubated for 30 minutes at room temperature. Three other washing cycles ensued, followed by a final demiwater wash to prevent interference from the washing buffer with the HRP detection. Tetramethylbenzidine (TMB) substrate was added for the HRP-detection and incubated for 30 minutes at room temperature in the dark. Positivity in the wells was indicated by blue coloration in the wells. This reaction was stopped using stop solution which changed the blue coloration to yellow. Signal readout was performed using an ELISA plate reader (Bio-Rad, Benchmark Plus microplate reader) at dual-wavelength 450/620 (substracted).

**Cell differentiation**

Differentiation rate of C212 myoblasts to myocytes was determined using staining techniques. After five days of addition of differentiation medium (DMEM, 2,5%; horse serum (HS, Life Technologies, ThermoFisher; Cat. No. 26050088), 1% ps), DFO with or without FC were added. Two days later, C2C12 cells were fixed with 4% formaldehyde during ten minutes and stained with anti-Myh3 (R&D systems, Mn, USA, Mab4470) as a marker of differentiated myocytes, using DAPI medium (Vectashield, Vector Laboratories, Ca, USA). Four to six different fields per well were captured randomly. The percentage of the number of nuclei inside Myh3-positive cells from the total number of counted nuclei per field was calculated and referred to as the fusion index.

***Seahorse mitochondrial flux analysis***

Oxygen consumption rate (OCR) and extracellular acidification rate (ECAR) of skeletal myocytes were measured using a Seahorse Mito Stress test. C2C12 myoblasts were seeded at 5000 cells/well or 1800 cells/well on a Seahorse 24-well or 96-well culture plate, respectively, and cultured with differentiation medium with or without DFO and FC as described above. Before running the assay, the cells were equilibrated by incubating them with XF medium (102365-100, Agilent) supplemented with 10 mM glucose (Sigma-Aldrich, Car. No. G7021), 1 mM sodium pyruvate (Thermo Fisher, Cat. No. 11360070) and 2 mM glutamine (Lonza, Cat. No. BE17-605E) for one hour at 37°C 0% CO_2_. During the assay in which the 24-well culture plate was used, three baseline measurements were taken, after which 1 µM of ATP synthase inhibitor Oligomycin A (Sigma-Aldrich, Cat. No. No 75351) was injected into the wells to inhibit complex I of the oxidative phosphorylation process. Then, 1 µM of carbonyl cyanide 4-(trifluoromethoxy)phenylhydrazone (FCCP, Sigma-Aldrich, Cat. No. No C2920) was injected to uncouple the oxidative phosphorylation and induce rapid oxidation of energy substrates. Finally, a mixture of 1µM rotenone (Sigma-Aldrich, Cat. No. R8875) and 1 µM antimycin A (Sigma-Aldrich, Cat. No. No A8674) was added to the wells to completely inhibit oxidative phosphorylation in the cells, allowing for the measurement of non-mitochondrial cellular oxygen consumption. For the last OCR assay and for the ECAR assay, a 96-well plate was used and data were acquired in a similar fashion, with 100 µM bromopyruvate (Sigma Aldrich, Cat. No. 16490-10G) as a fourth addition to completely inhibit glycolysis. All data were acquired using a Seahorse XF24 or XF96 analyzer and normalized for total protein in the well. Data were analyzed using Wave software from Agilent. To assess ATP-linked respiration, the decrease in oxygen consumption rate after the injection of oligomycin A was calculated (OCR_basal_ - OCR_oligomycin_). To assess respiratory reserve of the cells, the increase in oxygen consumption after injection of FCCP compared to baseline was calculated (OCR_FCCP_ – OCR_basal_).

***Western blotting***

C2C12 cell lysate was collected using Radioimmunoprecipitation assay (RIPA) buffer (Chem Cruz Cat. No. No sc24948) supplemented with protease inhibitors, sodium orthovanadate and phenylmethylsulfonyl fluoride (all provided with RIPA) through incubation on ice for ten minutes before centrifugation at 12,000 x g at 4°C for 10 minutes. Supernatant was collected and total protein concentration was measured using a Bicinchoninic acid (BCA) protein assay (ThermoScientific, Cat. No. 23227). Lysates were denaturized in 5x Laemmli samples buffer concentrate by incubating them at 95-100°C for 10 minutes in a heat block. Normalized protein amounts were loaded onto 4-20% Mini-Protean TGX precast protein gels (Bio-Rad Cat. No. 4561094) and SDS-PAGE was performed. Afterwards, the proteins in the gel were transferred to polyvinylidene fluoride (PVDF; Bio-Rad Cat. No. 1620177) membranes. Blocking of membranes was done by overnight incubation with 5% non-fat milk blocking buffer (Campina, ELK milk powder) at 4°C. The following day, primary antibody incubation was performed for one hour at room temperature with Ferritin Heavy Chain I (Fth) (Cell signaling Technology, Cat. No. 4393s), Beclin-1 (Cell signaling Technology, Cat. No. 3738s), Myoglobin (Abcam, Cat. No. ab77232) and ß-actin (Cell Signaling Technology, Cat. No. 4967s) rabbit antibodies diluted in 5% non-fat milk blocking buffer. The membranes were washed three times for five minutes with 0,1% tris-buffered saline-Tween-20 (TBS-T) in between antibody incubations. Secondary antibody goat- anti rabbit IgG Horseradish peroxidase (HRP)-linked (DAKO, Cat. No. P0448) was diluted in 1% non-fat milk blocking buffer and incubated with the membranes for one hour at room temperature. After secondary antibody incubation, membranes were washed twice with TBST for five minutes. One final wash with TBS was performed for five minutes before visualizing bands using chemiluminescence reagents (Perkin Elmer, Cat. No. NEL111001EA). Imaging of the blots was done using ChemiDocMP (Bio-Rad). All images were analyzed using Fiji-ImageJ software and signal intensities were normalized for β-actin.

***RNA extraction***

For quantitative real-time PCR and for RNA sequencing, total ribonucleic acid (RNA) was isolated from C2C12 myoblasts and myocytes using Favorprep tissue total RNA mini kit (Favorgen, Cat. No. FATRK) according to manufacturer’s protocol. RNA concentration was determined with Nanodrop measurements (Nanodrop, ND-1000). RNA samples were stored in RNAse-free water (Favorgen).

***Quantitative real-time PCR***

For quantitative real-time PCR, messenger RNA (mRNA) was isolated from C2C12 myocytes as described above. Equal amounts of complementary deoxyribonucleic acid (cDNA) were synthesized using Quantitect Reverse Transcription kit (Qiagen, Cat. No. 205311) which included a genomic DNA elimination step. Quantitative polymerase chain reaction (qPCR) technology was then performed by mixing collected cDNA with primers for target genes and SYBR green master mix (Thermo Fisher, Cat. No. 4385617). Mouse primers for genes *Fbxo32*, *Trim63*, *Becn1*, *TfR*, *Slc39a14 and Slc40a1* were bought on-demand (Sigma-Aldrich). Additionally, primers for *Bax*, *Bcl2* and *Casp9* were acquired (Supplemental Table 1). *RplpO* was quantified as housekeeping gene. All qPCR measurements were executed at 95°C for 10 minutes followed by 40 cycles at 95°C for 15 seconds and 60°C for 40 seconds. Specificity of the primers was determined by applying a melt curve analysis. Relative quantification of gene expression was done using the acquired Ct-values and applying the ∆∆Ct-method in Microsoft Excel. Experiments were performed on a Viia7 RT-PCR system (Applied Biosystems) and raw data was processed using Quantstudio RT-PCR software v1.3 (Applied Biosystems). The primer sequences are described in table S1.

***RNA sequencing***

For RNA sequencing, mRNA was isolated from C2C12 myoblasts and myocytes as described above. Samples containing at least 1 µg RNA (≥ 20 ng/µL, ≥ 5 µL) with a high purity (A260/2.80 2.0-2.2) were sent to Single Cell Discoveries (Utrecht, the Netherlands) for transcriptome analysis, for which an adapted version of the CEL-seq protocol was used. Samples were multiplexed into a sequencing library and sequenced. Total RNA concentration was measured and normalized to 20 ng/ul using a Qubit fluorometer (Invitrogen), and RNA quality was assessed via bioanalyzer and RNA Pico 6000 kit (Agilent). Normalized total RNA (with RNA integrity number (RIN) scores >7) was used for library preparation and sequencing. mRNA was processed as described previously, following an adapted version of the single-cell mRNA seq protocol of CEL-Seq[S6, S7]. In brief, samples were barcoded with CEL-seq primers during a reverse transcription and pooled after second strand synthesis. The resulting cDNA was amplified with an overnight in vitro transcription reaction. From this amplified RNA, sequencing libraries were prepared with Illumina Truseq small RNA primers. The DNA library was paired-end sequenced on an Illumina Nextseq™ 500, high output, with a 1x75 bp Illumina kit (R1: 26 cycles, index read: 6 cycles, R2: 60 cycles).

Read 1 was used to identify the Illumina library index and CEL-Seq sample barcode. Read 2 was aligned to the Mouse mm10 + mitochondrial genes reference transcriptome using BWA MEM[S8]. Reads that mapped equally well to multiple locations were discarded. Mapping and generation of count tables was done using the MapAndGo script1.

Differentially expressed genes (DEGs) were determined with the DESeq2 package in R[S9] by using a likehood ratio test to compare all conditions (i.e., control, DFO, and DFO + FC). These comparisons were made for myoblasts and myocytes separately or together. Pathway enrichment analysis was performed using GOrilla[S10-S11], and REVIGO[S12] to identify biological processes that were affected by ID and iron supplementation. Finally, computational prediction of putative transcription factors that might govern clusters of identified differentially expressed genes was done using ChIP-X Enrichment Analysis 3 (ChEA3)[S13]: the top 10 transcription factors were selected based on evidence from literature.

***Fluorescence-activated Cell Sorting***

To assess apoptosis rate in differentiated C2C12 myocytes, fluorescence-activated cell sorting (FACS) was used. C2C12 myoblasts were seeded at 100.000 cells/well and cultured for two days in culture medium. Next, medium was substituted with differentiation medium (DMEM, 2,5% HS, 1%). After five days, DFO and/or FC was pipetted into the plate prior to the last two days of incubation.

Afterwards, C2C12 cells were collected from the plate using 150µl 0,05% Trypsin-EDTA (Thermo Scientific, Cat. No. 15400-054). Cells were stained with 5µl FITC Annexin-V and 5µl propidium ionide staining solution (both BD Biosciences Cat. No. 556547) to distinguish early apoptotic cells and late apoptotic cells from living cells. Apoptosis rate was analyzed using the NovoCyte Queanteon (ACEA Biosciences). Acquired data were analyzed using NovoExpress.

***Supplementary methods references***

S1. Kieneker LM, Gansevoort RT, Mukamal KJ, de Boer RA, Navis G, Bakker SJ et al. Urinary potassium excretion and risk of developing hypertension: the prevention of renal and vascular end-stage disease study. Hypertension. 2014 Oct;64(4):769-76.

S2. Inker LA, Schmid CH, Tighiouart H, Eckfeldt JH, Feldman HI, Greene T et al. Estimating glomerular filtration rate from serum creatinine and cystatin C. N Engl J Med. 2012 Jul 5;367(1):20-9.

S3. Cockcroft DW, Gault MH. Prediction of creatinine clearance from serum creatinine. Nephron. 1976;16(1):31-41.

S4. Groothof D, Post A, Polinder-Bos HA, Erler NS, Flores-Guerrero JL, Kootstra-Ros JE et al. Muscle mass and estimates of renal function: a longitudinal cohort study. J Cachexia Sarcopenia Muscle. 2022 Aug;13(4):2031-2043.

S5. van Vliet IMY, Post A, Kremer D, Boslooper-Meulenbelt K, van der Veen Y, de Jong MFC et al. Muscle mass, muscle strength and mortality in kidney transplant recipients: results of the TransplantLines Biobank and Cohort Study. J Cachexia Sarcopenia Muscle. 2022 Oct.

S6. Hashimshony T, Wagner F, Sher N, Yanai I. CEL-Seq: single-cell RNA-Seq by multiplexed linear amplification. Cell Rep. 2012 Sep 27;2(3):666-73.

S7. Simmini S, Bialecka M, Huch M, Kester L, van de Wetering M, Sato T, Beck F, van Oudenaarden A, Clevers H, Deschamps J. Transformation of intestinal stem cells into gastric stem cells on loss of transcription factor Cdx2. Nat Commun. 2014 Dec 11;5:5728.

S8. Li H, Durbin R. Fast and accurate long-read alignment with Burrows-Wheeler transform. Bioinformatics. 2010 Mar 1;26(5):589-95.

S9. Love MI, Huber W, Anders S. Moderated estimation of fold change and dispersion for RNA-seq data with DESeq2. Genome Biol. 2014;15(12):550.

S10. Eden E, Navon R, Steinfeld I, Lipson D, Yakhini Z. GOrilla: a tool for discovery and visualization of enriched GO terms in ranked gene lists. BMC Bioinformatics. 2009 Feb 3;10:48.

S11. Eden E, Lipson D, Yogev S, Yakhini Z. Discovering motifs in ranked lists of DNA sequences. PLoS Comput Biol. 2007 Mar 23;3(3):e39.

S12. Supek F, Bošnjak M, Škunca N, Šmuc T. REVIGO summarizes and visualizes long lists of gene ontology terms. PLoS One. 2011;6(7):e21800.

S13. Keenan AB, Torre D, Lachmann A, Leong AK, Wojciechowicz ML, Utti V, Jagodnik KM, Kropiwnicki E, Wang Z, Ma'ayan A. ChEA3: transcription factor enrichment analysis by orthogonal omics integration. Nucleic Acids Res. 2019 Jul 2;47(W1):W212-W224.

1. **Supplemental Tables**

| **Gene** | **Forward sequence** | **Reverse sequence** |
| --- | --- | --- |
| *Fbxo32* | 5’-ACTCTCTACACATCCTTATGC-3’ | 5’-TGATGTTCAGTTGTAAGCAC-3’ |
| *Trim63* | 5’-GGGTAAAGAAGAACACCAATG-3’ | 5’-GAAGACACACTTCCCTATTG-3’ |
| *Becn1* | 5’-TAGTGAGTTTAAAAGGCAGC-3’ | 5’-CGACCCAGTCTGAAATTATTG-3’ |
| *TfR* | 5’-CAGAAGTTTCTGGTAAACTGG-3’ | 5’-TCTGCAAAAGTAATTTCCCC-3’ |
| *Slc39a14* | 5’-AGAATGAACAGACAGAGGAG-3’ | 5’-AAGGCTATGAAGTAGAGCAG-3’ |
| *Slc40a1* | 5’-TCACCTAAAGATACTGAGCC-3’ | 5’-CTGGTTATAGTAGGAGACCC-3’ |
| *Myh7* | 5’-GATGATCTATACCTACTCGGG-3’ | 5’-TGATGAGGATGGACTGATTC-3’ |
| *Myod* | 5’-CTGTGTAGTGCAACAAAAAC-3’ | 5’-TTTATTTCCAACACCTGAGC-3’ |
| *Myog* | 5’-CCCATTCACATAAGGCTAAC-3’ | 5’-CACTGAGGGACATTAACAAG-3’ |
| *Bax* | 5’-ACTAAAGTGCCCGAGCTGAT-3’ | 5’- ATGGTCACTGTCTGCCATGT-3’ |
| *Bcl2* | 5’-GTGGATGACTGAGTACCTGA-3’ | 5’-AAACAGAGGTCGCATGCT-3’ |
| *Casp9* | 5’-TTCCCAGGTTTTGTCTCCTG-3’ | 5’-GGGACTGCAGGTCTTCAGAG-3’ |
| *Rplp0* | 5’- AAGCGCGTCCTGGCATTGTC-3’ | 5’-GCAGCCGCAAATGCAGATGG-3 |

**Supplemental Table 1.** Mouse primers for RT-PCR.

|  | **Myoblasts** | **Myocytes** |
| --- | --- | --- |
| **1** | HIF1A | FOXM1 |
| **2** | E2F1 | E2F4 |
| **3** | RUNX2 | HIF1A |
| **4** | TCF21 | E2F1 |
| **5** | KLF1 | MYBL2 |
| **6** | POU5F1 | MYC |
| **7** | HSF1 | AR |
| **8** | GATA2 | KLF4 |
| **9** | MYC | KLF1 |
| **10** | GATA6 | NR1H3 |

**Supplemental Table 2.** Top ten transcription factors known, based on literature, to be involved in expression of the largest number of differentially expressed genes after induction of iron deficiency.

1. **Supplemental Figures**

**Figure legends**

**Supplemental Figure 1**. Flowchart of inclusion

**Supplemental Figure 2. Differentiation of C2C12 myoblasts to myocytes after addition of differentiation medium.** Gene expression of differentiation markers Myh7 (A), Myod (B) and Myog (C) before and after seven days of culturing with differentiation medium. Measurements are normalized for housekeeping gene expression and for untreated controls. (D) Fusion index before and after seven days of culturing with differentiation medium. Data are based on three separate experiments each.

**Supplemental Figure 3**. **Association between iron status as reflected by ferritin levels (A, C) or TSAT (B, D) and CER indexed for length (A, B) or CER indexed for length squared (C, D) in community-dwelling individuals.** Odds ratios and corresponding 95% confidence intervals are provided for the risk of being in the lowest age- and sex-specific quintile of 24-hour CER in a crude model (Model 1), a multivariable model, adjusted for BMI, eGFR, hs-CRP, urinary urea excretion, alcohol consumption and smoking status (Model 2) and with additional adjustment for hemoglobin (Model 3).

Abbreviations: CER, creatinine excretion rate; TSAT, transferrin saturation; eGFR, estimated glomerular filtration rate; BMI, body mass index; hs-CRP, high sensitive C-reactive protein; OR, odds ratio.

**Supplemental Figure 4**. **Association between iron status as reflected by ferritin levels (A) or TSAT (B) and CER in community-dwelling individuals, after excluding the 5% most extreme outliers of ferritin (A) or TSAT (B).** Odds ratios and corresponding 95% confidence intervals are provided for the risk of being in the lowest age- and sex-specific quintile of 24-hour CER in a crude model (Model 1), a multivariable model, adjusted for BMI, eGFR, hs-CRP, urinary urea excretion, alcohol consumption and smoking status (Model 2) and with additional adjustment for hemoglobin (Model 3).

Abbreviations: CER, creatinine excretion rate; TSAT, transferrin saturation; eGFR, estimated glomerular filtration rate; BMI, body mass index; hs-CRP, high sensitive C-reactive protein; OR, odds ratio.

**Supplemental Figure 5. Induction of intracellular ID by DFO is reversed by FC in C2C12 myoblasts.** Protein quantification of Fth in myoblasts assessed with Western Blot analysis after incubation with or without DFO without (A) or with (B) FC for three days. All measurements are normalized for total protein content, for β-actin content and for untreated controls. Experiments performed in myoblasts are depicted as closed circles. Data are based on three separate experiments each. Experiments performed without DFO or FC are depicted as black symbols, experiments with DFO as red symbols and experiments with DFO and FC as blue symbols.

**Supplemental Figure 6. Treatment of C2C12 myoblasts with DFO leads to impaired proliferation rate.** Proliferation rate was assessed with a BrdU cell proliferation ELISA assay under increasing concentrations of DFO. Data are based on three separate experiments each. Experiments performed without DFO or FC are depicted as black symbols, experiments with DFO as red symbols and experiments with DFO and FC as blue symbols.

**Supplemental Figure 7. Treatment with 7.5 µM deferoxamine (DFO) may induce apoptosis.** Percentage of apoptotic or necrotic cells assessed with flow cytometry techniques. Data are based on three separate experiments each. Experiments performed without DFO or FC are depicted as black symbols, experiments with DFO as red symbols and experiments with DFO and FC as blue symbols.

**Supplemental Figure 8.** Revigo TreeMap of Gene Ontology (GO) terms reflecting biological processes in which the differentially expressed genes under deferoxamine treatment in myoblasts (**A)** and myocytes (**B**) are involved. Each rectangle represents a GO term and related terms are combined into clusters with the same color. The size of the rectangles represents the frequency of the GO term related to the differentially expressed genes as well as the P-value.
