# Supplementary figures and images for: Iron deficiency is related to lower muscle mass in community‐dwelling individuals and impairs myoblast proliferation
Source: J Cachexia Sarcopenia Muscle. 2023 Jun 30;14(4):1865–79. doi: 10.1002/jcsm.13277 (PMC10401536; doi:10.1002/jcsm.13277)

Flowchart of inclusion

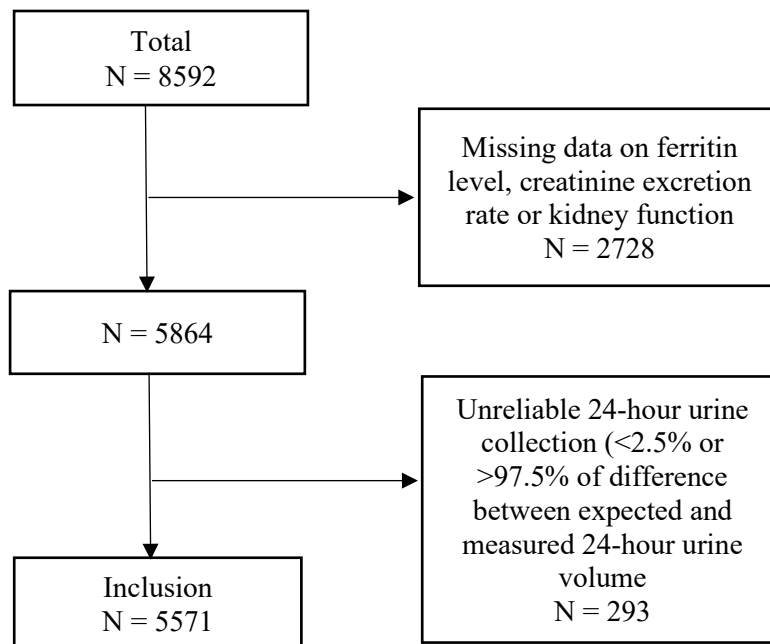

Supplement: Supplementary file 2 — Figure S1. Flowchart of inclusion. [file JCSM-14-1865-s006.pdf]

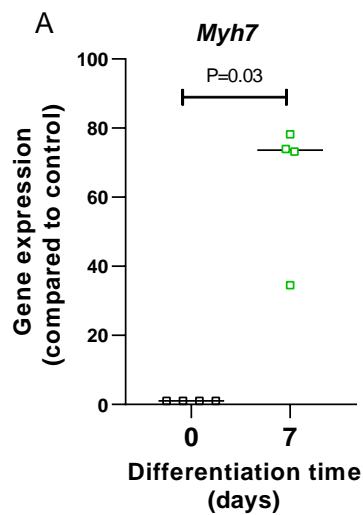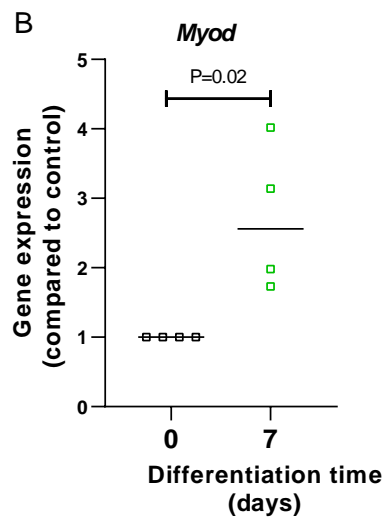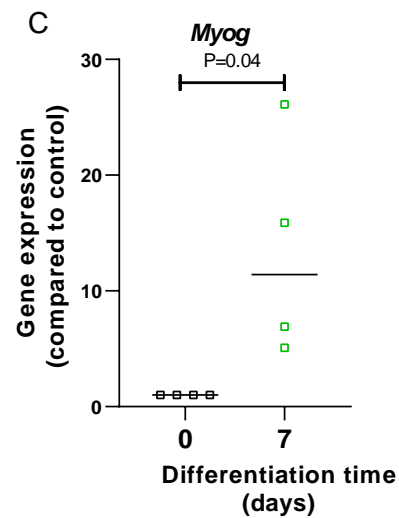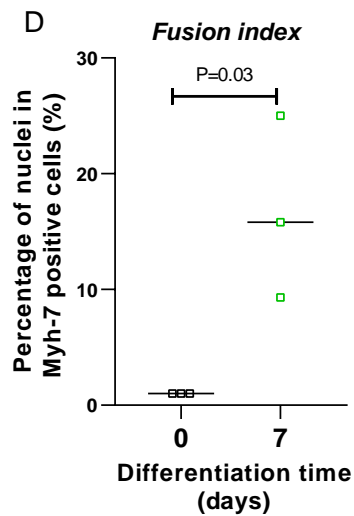

Supplement: Supplementary file 3 — Figure S2. Differentiation of C2C12 myoblasts to myocytes after addition of differentiation medium. Gene expression of differentiation markers Myh7 (A), Myod (B) and Myog (C) before and after seven days of culturing with differentiation medium. Measurements are normalized for housekeeping gene expression and for untreated controls. (D) Fusion index before and after seven days of culturing with differentiation medium. Data are based on three separate experiments each. [file JCSM-14-1865-s004.pdf]

### Ferritin & creatinine excretion rate (CER)

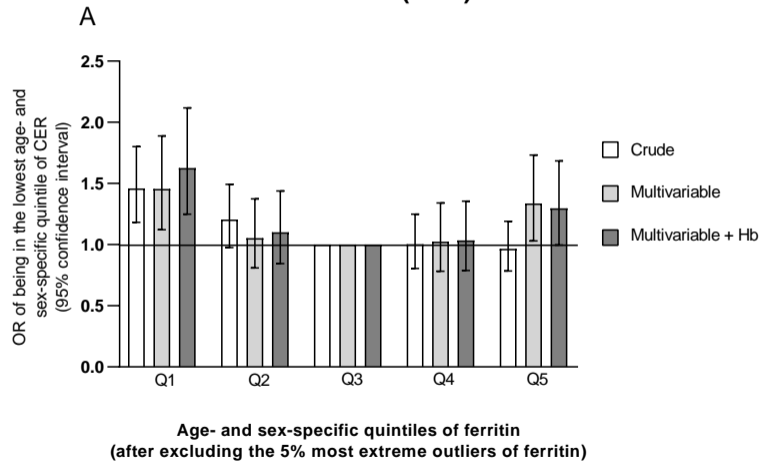

### TSAT & creatinine excretion rate (CER)

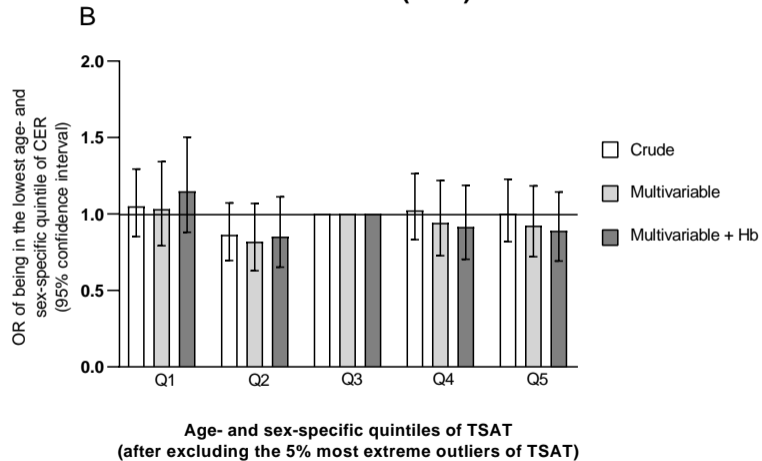

Supplement: Supplementary file 5 — Figure S4. Association between iron status as reflected by ferritin levels (A) or TSAT (B) and CER in community‐dwelling individuals, after excluding the 5% most extreme outliers of ferritin (A) or TSAT (B). Odds ratios and corresponding 95% confidence intervals are provided for the risk of being in the lowest age‐ and sex‐specific quintile of 24‐hour CER in a crude model (Model 1), a multivariable model, adjusted for BMI, eGFR, hs‐CRP, urinary urea excretion, alcohol consumption and smoking status (Model 2) and with additional adjustment for haemoglobin (Model 3). Abbreviations: CER, creatinine excretion rate; TSAT, transferrin saturation; eGFR, estimated glomerular filtration rate; BMI, body mass index; hs‐CRP, high sensitive C‐reactive protein; OR, odds ratio. [file JCSM-14-1865-s002.pdf]

A

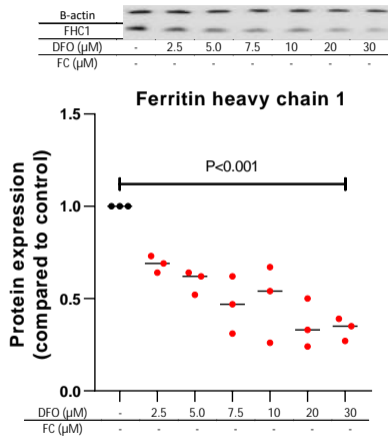

B

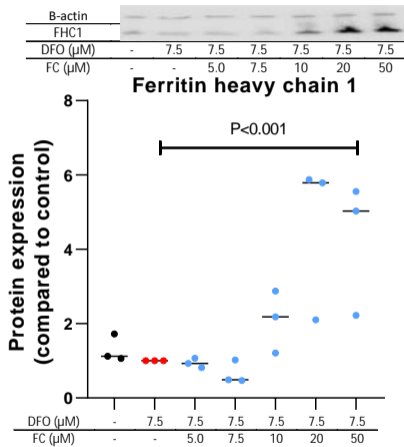

Supplement: Supplementary file 6 — Figure S5. Induction of intracellular ID by DFO is reversed by FC in C2C12 myoblasts. Protein quantification of Fth in myoblasts assessed with Western Blot analysis after incubation with or without DFO without (A) or with (B) FC for three days. All measurements are normalized for total protein content, for β‐actin content and for untreated controls. Experiments performed in myoblasts are depicted as closed circles. Data are based on three separate experiments each. Experiments performed without DFO or FC are depicted as black symbols, experiments with DFO as red symbols and experiments with DFO and FC as blue symbols. [file JCSM-14-1865-s009.pdf]

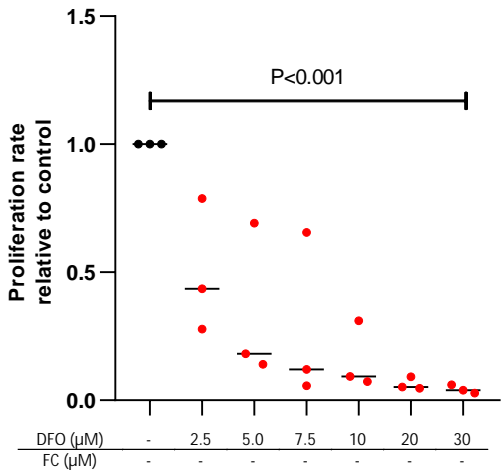

Supplement: Supplementary file 7 — Figure S6. Treatment of C2C12 myoblasts with DFO leads to impaired proliferation rate. Proliferation rate was assessed with a BrdU cell proliferation ELISA assay under increasing concentrations of DFO. Data are based on three separate experiments each. Experiments performed without DFO or FC are depicted as black symbols, experiments with DFO as red symbols and experiments with DFO and FC as blue symbols. [file JCSM-14-1865-s007.pdf]

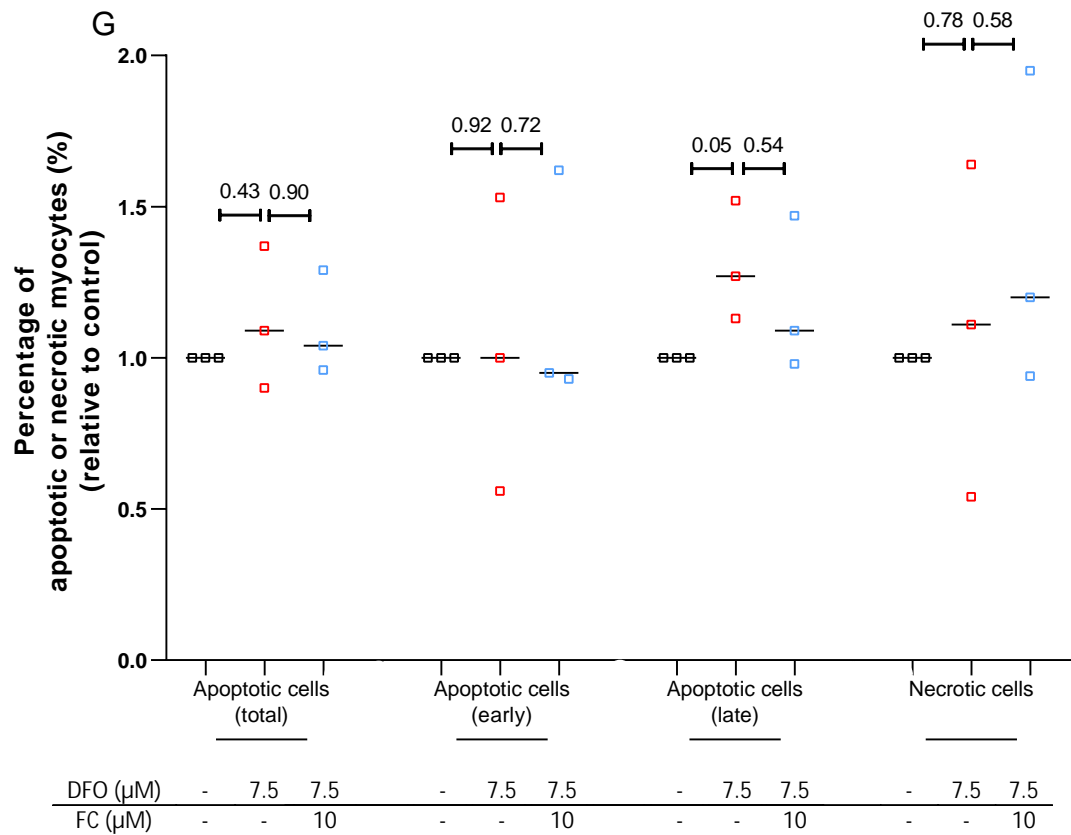

Supplement: Supplementary file 8 — Figure S7. Treatment with 7.5 μM deferoxamine (DFO) may induce apoptosis. Percentage of apoptotic or necrotic cells assessed with flow cytometry techniques. Data are based on three separate experiments each. Experiments performed without DFO or FC are depicted as black symbols, experiments with DFO as red symbols and experiments with DFO and FC as blue symbols. [file JCSM-14-1865-s005.pdf]

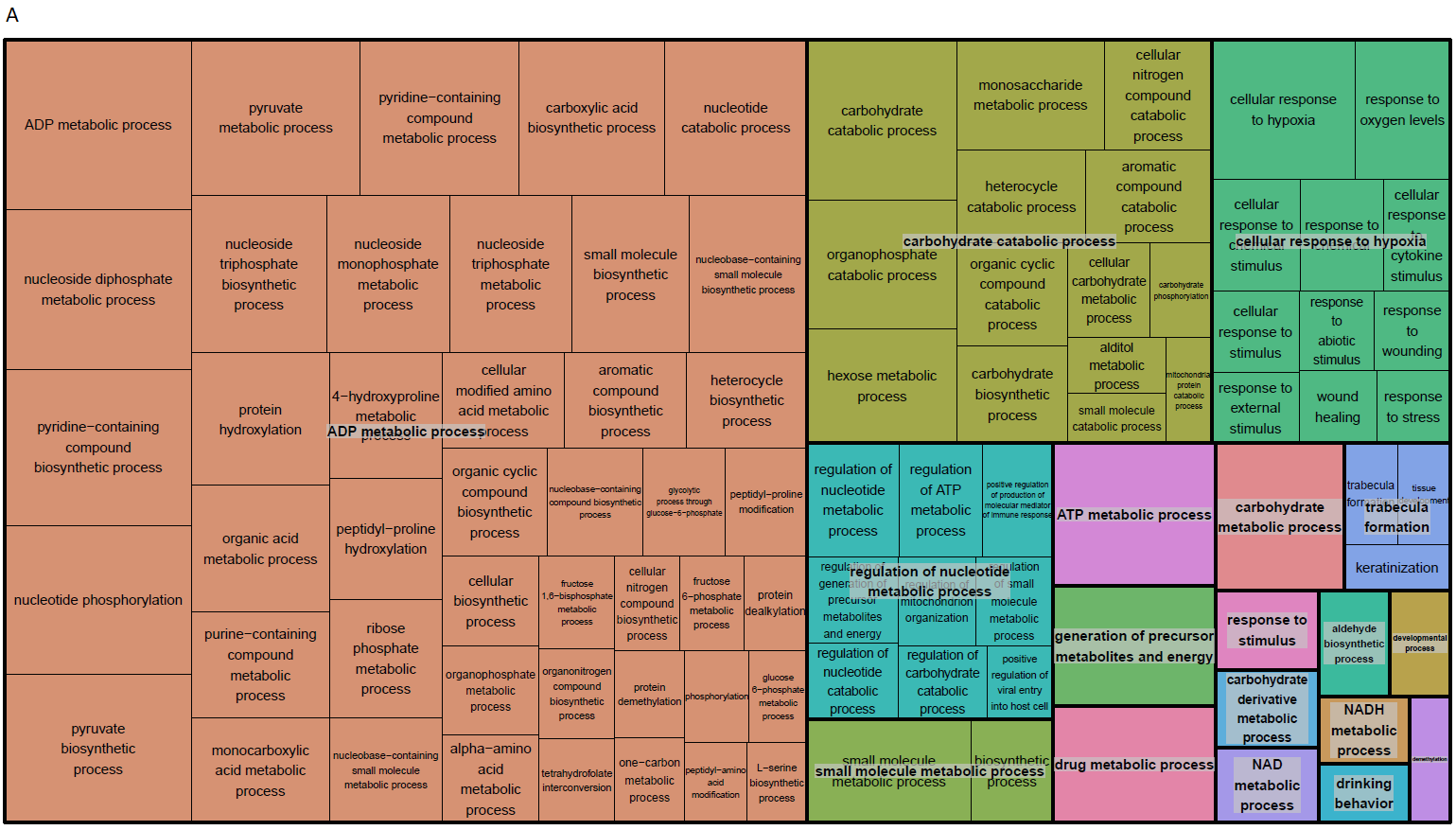

Supplement: Supplementary file 9 — Figure S8. Revigo TreeMap of Gene Ontology (GO) terms reflecting biological processes in which the differentially expressed genes under deferoxamine treatment in myoblasts (A) and myocytes (B) are involved. Each rectangle represents a GO term and related terms are combined into clusters with the same colour. The size of the rectangles represents the frequency of the GO term related to the differentially expressed genes as well as the P‐value. [file JCSM-14-1865-s003.zip › Supplemental Figure 8a.png]

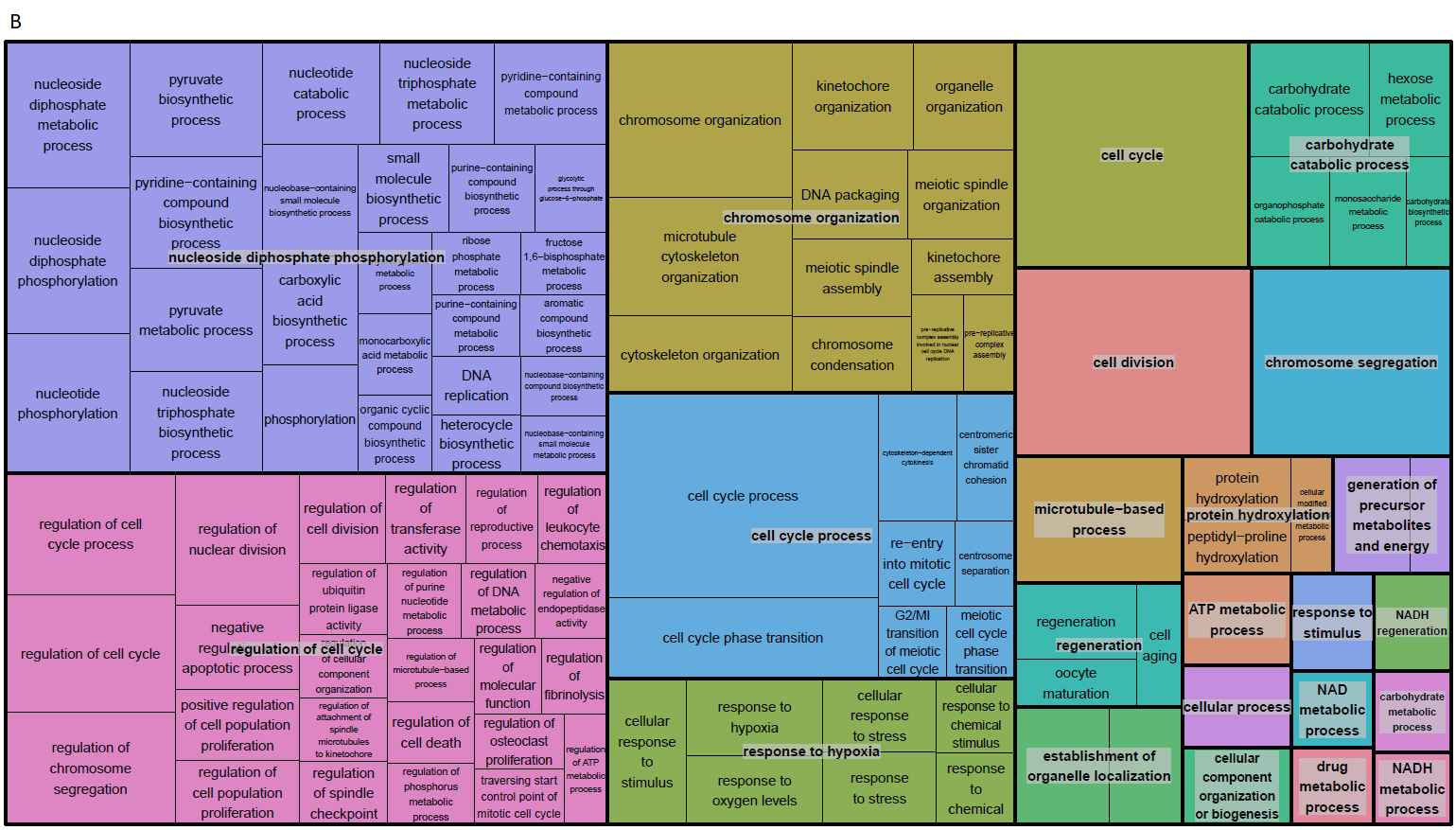

Supplement: Supplementary file 9 — Figure S8. Revigo TreeMap of Gene Ontology (GO) terms reflecting biological processes in which the differentially expressed genes under deferoxamine treatment in myoblasts (A) and myocytes (B) are involved. Each rectangle represents a GO term and related terms are combined into clusters with the same colour. The size of the rectangles represents the frequency of the GO term related to the differentially expressed genes as well as the P‐value. [file JCSM-14-1865-s003.zip › Supplemental Figure 8b.png]
