# Supplementary material for: Iron deficiency is related to lower muscle mass in community‐dwelling individuals and impairs myoblast proliferation
Source: J Cachexia Sarcopenia Muscle. 2023 Jun 30;14(4):1865–79. doi: 10.1002/jcsm.13277 (PMC10401536; doi:10.1002/jcsm.13277)
Supplement: Supplementary file 4 — Figure S3. Association between iron status as reflected by ferritin levels (A, C) or TSAT (B, D) and CER indexed for length (A, B) or CER indexed for length squared (C, D) in community‐dwelling individuals. Odds ratios and corresponding 95% confidence intervals are provided for the risk of being in the lowest age‐ and sex‐specific quintile of 24‐hour CER in a crude model (Model 1), a multivariable model, adjusted for BMI, eGFR, hs‐CRP, urinary urea excretion, alcohol consumption and smoking status (Model 2) and with additional adjustment for haemoglobin (Model 3). Abbreviations: CER, creatinine excretion rate; TSAT, transferrin saturation; eGFR, estimated glomerular filtration rate; BMI, body mass index; hs‐CRP, high sensitive C‐reactive protein; OR, odds ratio. [file JCSM-14-1865-s001.pdf]

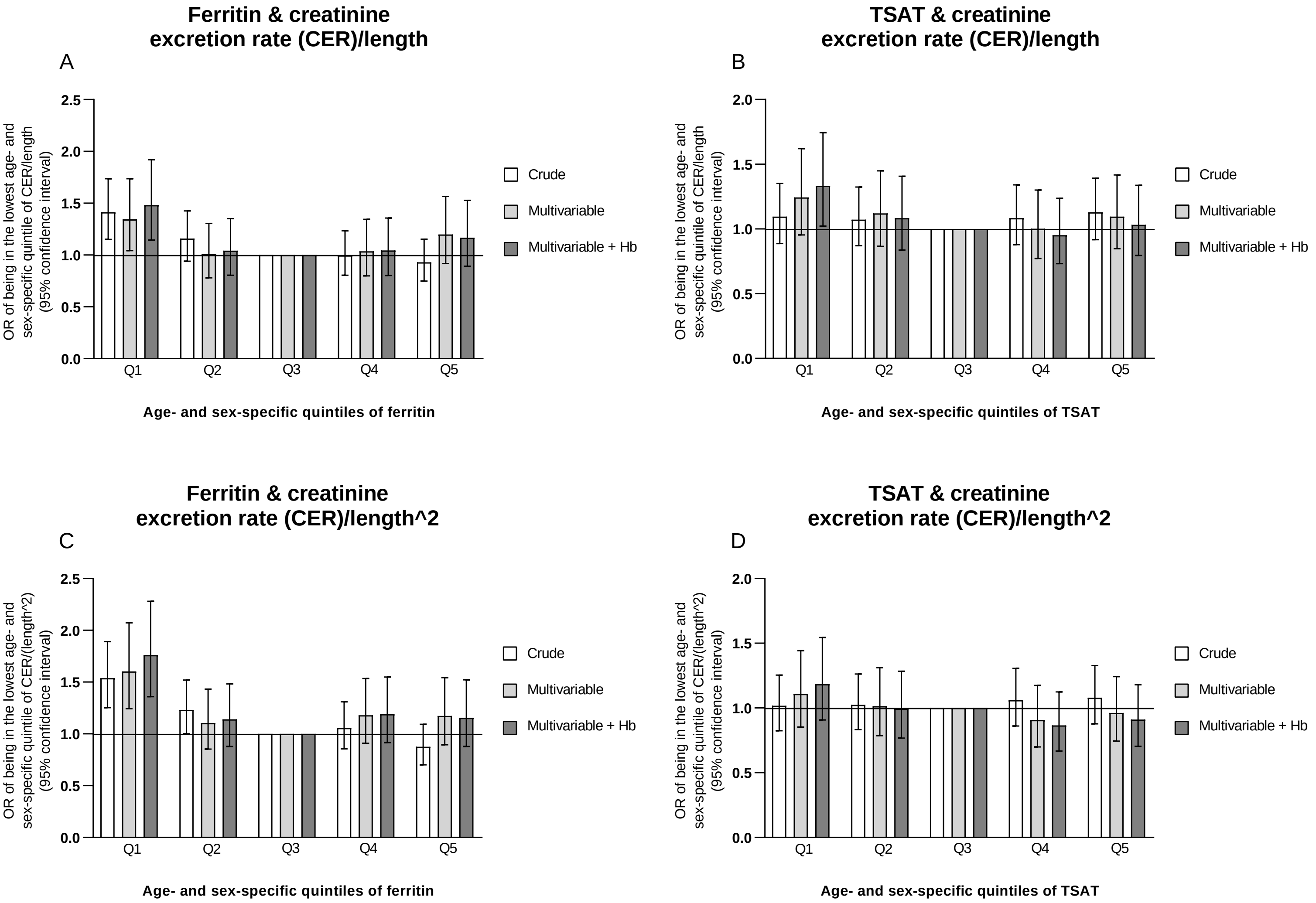
Ferritin & creatinine excretion rate (CER)/length
A
OR of being in the lowest age- and sex-specific quintile of CER/length (95% confidence interval)
2.5
2.0
1.5
1.0
0.5
0.0
Q1
Q2
Q3
Q4
Q5
Age- and sex-specific quintiles of ferritin
Crude
Multivariable
Multivariable + Hb
TSAT & creatinine excretion rate (CER)/length
B
OR of being in the lowest age- and sex-specific quintile of CER/length (95% confidence interval)
2.0
1.5
1.0
0.5
0.0
Q1
Q2
Q3
Q4
Q5
Age- and sex-specific quintiles of TSAT
Crude
Multivariable
Multivariable + Hb
Ferritin & creatinine excretion rate (CER)/length^2
C
OR of being in the lowest age- and sex-specific quintile of CER/(length^2) (95% confidence interval)
2.5
2.0
1.5
1.0
0.5
0.0
Q1
Q2
Q3
Q4
Q5
Age- and sex-specific quintiles of ferritin
Crude
Multivariable
Multivariable + Hb
TSAT & creatinine excretion rate (CER)/length^2
D
OR of being in the lowest age- and sex-specific quintile of CER/(length^2) (95% confidence interval)
2.0
1.5
1.0
0.5
0.0
Q1
Q2
Q3
Q4
Q5
Age- and sex-specific quintiles of TSAT
Crude
Multivariable
Multivariable + Hb
